# Proteolytic processing of galectin-3 by meprin metalloproteases is crucial for host-microbiome homeostasis

**DOI:** 10.1126/sciadv.adf4055

**Published:** 2023-03-31

**Authors:** Cynthia Bülck, Elisabeth E. L. Nyström, Tomas Koudelka, Michael Mannbar-Frahm, Gerrit Andresen, Mariem Radhouani, Florian Tran, Franka Scharfenberg, Friederike Schrell, Fred Armbrust, Eileen Dahlke, Bei Zhao, Alex Vervaeke, Franziska Theilig, Philip Rosenstiel, Philipp Starkl, Stephan P. Rosshart, Helmut Fickenscher, Andreas Tholey, Gunnar C. Hansson, Christoph Becker-Pauly

**Affiliations:** ^1^ Institute of Biochemistry, University of Kiel, 24118 Kiel, Germany.; ^2^ Institute of Experimental Medicine, University of Kiel, 24188 Kiel, Germany.; ^3^ Institute of Infection Medicine, University of Kiel and University Medical Center Schleswig-Holstein, 24015 Kiel, Germany.; ^4^ Division of Infection Biology, Department of Medicine I, Medical University of Vienna, 1090 Vienna, Austria.; ^5^ Institute of Clinical Molecular Biology, Kiel University and University Medical Center Schleswig-Holstein, 24105 Kiel, Germany.; ^6^ Institute of Anatomy, University of Kiel, 24118 Kiel, Germany.; ^7^ Department of Microbiome Research, Friedrich-Alexander-University Erlangen-Nürnberg, 91054 Erlangen, Germany.; ^8^ Department of Medicine II (Gastroenterology, Hepatology, Endocrinology, and Infectious Diseases), Medical Center–University of Freiburg, Faculty of Medicine, University of Freiburg, 79106 Freiburg, Germany.; ^9^ Department of Medical Biochemistry and Cell Biology, University of Gothenburg, 405 30 Gothenburg, Sweden.

## Abstract

The metalloproteases meprin α and meprin β are highly expressed in the healthy gut but significantly decreased in inflammatory bowel disease, implicating a protective role in mucosal homeostasis. In the colon, meprin α and meprin β form covalently linked heterodimers tethering meprin α to the plasma membrane, therefore presenting dual proteolytic activity in a unique enzyme complex. To unravel its function, we applied N-terminomics and identified galectin-3 as the major intestinal substrate for meprin α/β heterodimers. Galectin-3–deficient and meprin α/β double knockout mice show similar alterations in their microbiome in comparison to wild-type mice. We further demonstrate that meprin α/β heterodimers differentially process galectin-3 upon bacterial infection, in germ-free, conventionally housed (specific pathogen–free), or wildling mice, which in turn regulates the bacterial agglutination properties of galectin-3. Thus, the constitutive cleavage of galectin-3 by meprin α/β heterodimers may play a key role in colon host-microbiome homeostasis.

## Teaser

The intestinal microbiome influences galectin-3 proteolysis, which in turn modulates bacterial agglutination and composition.

## INTRODUCTION

The entire gastrointestinal tract is covered by a mucus layer that protects the intestinal epithelium against potential toxic compounds and shields the epithelium from commensal and pathogenic bacteria (*1*, *2*). Both an intact mucus layer and host-microbiome interactions are critical for health and disease and are related to infection and inflammation such as inflammatory bowel disease (IBD) (*3*). The metalloproteases meprin β and, particularly, meprin α are highly abundant in the gut, and the expression of *mep1a* is suggested as a marker gene for mature distal enterocytes (*4*). However, meprins are significantly decreased in inflamed intestinal tissue of patients with IBD (*5*–*7*). This could implicate that the expression of meprin metalloproteases in the gut has a protective biological function for mucosal and microbiome homeostasis. In the small intestine, meprin β is responsible for cleavage and detachment of the major mucus component mucin-2 (MUC2), thereby preventing bacterial overgrowth and infection (*8*, *9*). Proteolysis of MUC2 by meprin β is stimulated by the microbiome, supported by the observation that germ-free mice exhibit mucus strongly attached and accumulated to the epithelium (*8*). Consequently, the host-microbiome interaction seems to play an important role in the proteolysis-driven mucosal homeostasis. Although the function of meprin β in the small intestine is well characterized, the exact function of the protease and its close relative meprin α in the large intestine remains elusive. Meprin α and meprin β are zinc endopeptidases of the astacin family of the metzincin superfamily and are apart from the intestine also highly expressed in the kidney brush boarder (*4*, *10*–*12*). Meprin α and meprin β are heavily glycosylated multidomain type I transmembrane proteins. The main structural difference between meprin α and meprin β is the so-called “inserted” domain in meprin α, which contains a furin cleavage site. Meprin α is thus constitutively cleaved by furin in the secretory pathway, resulting in secretion of the protein. Homodimers of meprin α can combine to form noncovalent oligomers after secretion (*13*–*15*), whereas the meprin β dimer is predominantly membrane-bound on the cell surface. However, the inactive proform of meprin β can also be shed from the cell surface by a disintegrin and metalloprotease 10 (ADAM) and ADAM17 as well as membrane type 1 matrix metalloproteinase (MT1-MMP) (*16*–*19*). When meprin α and meprin β are coexpressed, particularly in the colon, they form a covalently linked heterodimeric complex in the early endoplasmic reticulum, which is transported to the cell surface, retaining meprin α on the plasma membrane (*7*). However, the functional consequence and the intestinal substrates for the meprin α/β heterodimer remain elusive.

To unravel the intestinal role of meprin α/β heterodimers with regard to chronic colitis, we aimed to identify intestinal substrates for this unique enzyme complex. Via mass spectrometry (MS)–based N-terminomics, we identified galectin-3 as a colonic substrate for the meprin α/β heterodimers. Notably, *lgals3*, such as *mep1a* and *mep1b,* is described as a marker gene for enterocytes (*4*). Galectin-3 is a 31-kDa chimeric-type member of the growing family of soluble β-galactoside binding lectin proteins (*20*). Among all galectins, galectin-3 is structurally unique consisting of a nonlectin N-terminal tail containing proline/glycine-rich repeating motifs, which facilitates its multimerization and a C-terminal carbohydrate recognition domain (CRD) (*21*).

Within the intestinal tract, galectin-3 is predominantly expressed in enterocytes of the villus tips (*22*) and is described to interact with MUC2 (*23*). Galectin-3 has also been demonstrated to interact with and agglutinate commensal and pathogenic bacteria, which provides evidence that galectin-3 may be important for host-microbiome homeostasis (*24*–*27*). As reported for meprins, decreased expression of galectin-3 is associated with IBD (*28*–*30*). In addition, galectin-3 knockout mice suffered from a more severe disease progression in a dextran sulfate sodium (DSS)–induced colitis model (*31*).

In this study, we demonstrate that galectin-3 undergoes constitutive proteolytic processing by the meprin α/β heterodimer in colonic mucosa and that this processing alters depending on bacterial load and composition. Furthermore, we show that cleavage of galectin-3 by meprin α/β heterodimers regulates bacterial agglutination and is crucial for the composition of the gut microbiome.

## RESULTS

### N-terminomics analysis identified galectin-3 as a colonic substrate for meprin α/β heterodimers

**Fig. 1.**
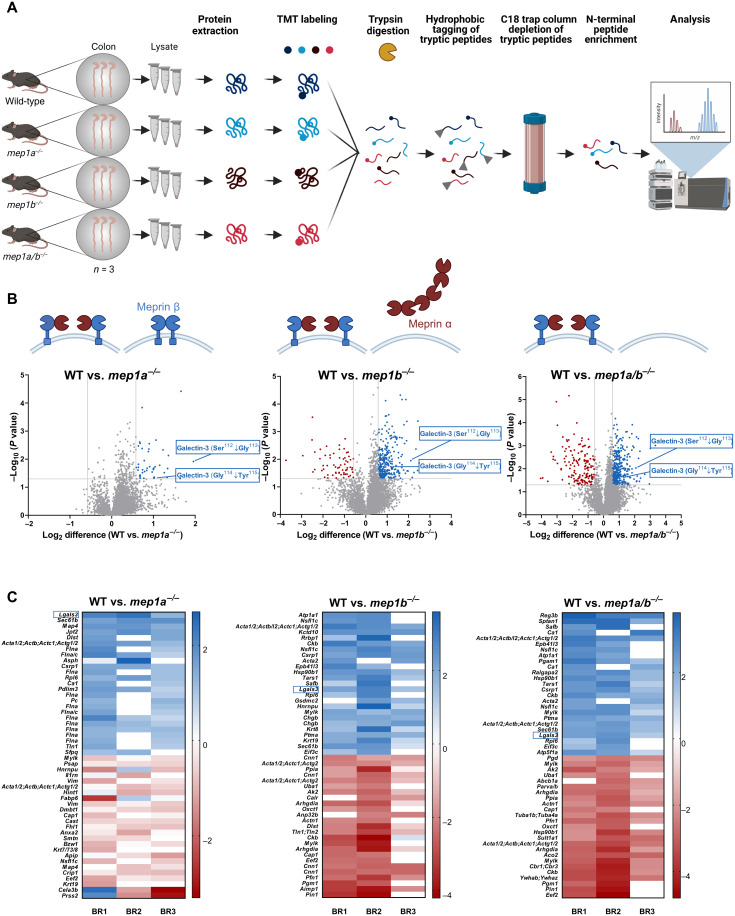
HYTANE analysis revealed galectin-3 as a major intestinal substrate for meprin α/β heterodimers. (**A**) For MS-based HYTANE analysis for the enrichment of N-terminal peptides tissue lysates of the proximal and distal colon from wild-type, meprin α knockout (*mep1a^−/−^*), meprin β knockout (*mep1b^−/−^*), and meprin α and meprin β double knockout (*mep1a/b^−/−^*) mice were used (*n* = 3). (**B**) Volcano plots showing all identified proteolytic events detected following HYTANE analysis of wild-type mice in comparison to *mep1a^−/−^*, *mep1b^−/−^*, or *mep1a/b^−/−^* mice. Gray lines represent threshold values (±0.58 for log_2_ difference and *P* = 0.05). Proteolytic fragments of galectin-3 could be identified in all approaches, indicating cleavage events between Ser^112^ and Gly^113^ or Gly^114^ and Tyr^115^. (**C**) Heatmaps of the top 25 highest (blue) and less (red) abundant proteolytic peptides from three biological replicates (BRs) (blue, wild-type > knockout; red, wild-type < knockout; sorted by log_2_ difference). In all approaches, cleavage of galectin-3 is more abundant in wild-type mice in comparison to the protease knockout mice (highlighted with a blue box).

To elucidate the physiological function of the meprin α/β heterodimers within the gut, we performed N-terminomics by MS-based HYTANE (hydrophobic tagging-assisted N termini enrichment) analysis of colon samples excluding the caecum from wild-type mice, meprin α knockout (*mep1a^−/−^)*, meprin β knockout (*mep1b^−/−^*), and meprin α and meprin β double knockout (*mep1a/b^−/−^*) mice (Fig. 1A) and screened for putative previously unknown proteolytic substrates. For meprin α/β heterodimers, 492 highly confident proteolytic cleavage events were identified (Fig. 1, B and C), which were associated with neutrophil degranulation or the adaptive immune system as revealed by Metascape analysis (fig. S1, A to C) (*32*). As we were particularly interested in substrates that are cleaved by the meprin α/β heterodimers, we selected for the high confident proteomic hits that were identified in the meprin single knockout and in the double knockout mice. The meprin α/β heterodimer is localized at the apical cell surface of enterocytes. We therefore sorted the identified N termini for extracellular substrates. Furthermore, we are predominantly interested into mucosal biology and its impact on the epithelial-microbiome interface. On the basis of these criteria, the best candidate substrate was galectin-3 (Fig. 1, B and C). In conclusion, our N-terminomics data revealed that galectin-3 is cleaved by meprins within its N-terminal tail near the C-terminal CRD (amino acids 132 to 264) between Ser^112^ and Gly^113^ as well as between Gly^114^ and Tyr^115^.

### Meprin α/β heterodimers constitutively cleave galectin-3 in the colon

**Fig. 2.**
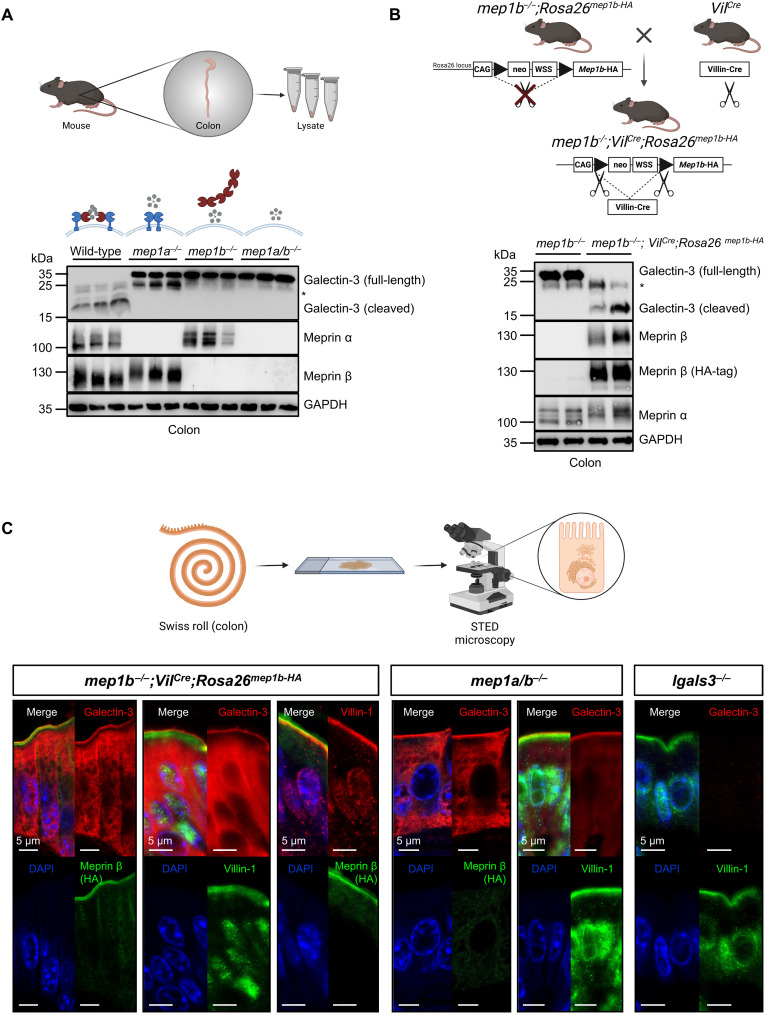
Proteolytic processing and tissue distribution of galectin-3 in mouse colon. (**A**) Galectin-3 cleavage in colonic tissue from three biological replicates of wild-type, *mep1a^−/−^*, *mep1b^−/−^*, or *mep1a/b^−/−^* mice analyzed by Western blot using a specific galectin-3, meprin α, and meprin β antibody. Glyceraldehyde-3-phosphate dehydrogenase (GAPDH) served as loading control. Galectin-3 was only proteolytically processed in wild-type mice when meprin α/β heterodimers are present in the colon. Asterisk marks an unspecific band. (**B**) Scheme of the generation of the *mep1b^−/−^;Vil^Cre^*;*Rosa26^mep1b-HA^* mice having an overall meprin β knockout with reexpressed meprin β–HA in enterocytes of the small and large intestines and Western blot analysis of colonic tissue of *mep1b^−/−^* and *mep1b^−/−^;Vil^Cre^*;*Rosa26^mep1b-HA^* mice to visualize galectin-3 cleavage via a specific galectin-3 antibody. Meprin β was either detected using a specific meprin β antibody or an HA-tag antibody against meprin β C terminus. GAPDH served as a loading control. Asterisk marks an unspecific band. (**C**) Stimulated emission depletion (STED) microscopy of intestinal Swiss roles from the whole colon of *mep1b^−/−^;Vil^Cre^*;*Rosa26^mep1b-HA^* mice, *mep1a/b^−/−^* mice, or *lgals3^−/−^* mice. Tissues were stained against galectin-3 (red), meprin β (green), or villin-1 (red or green). For reexpressed HA-tagged meprin β, the HA-antibody was used. Nuclear staining was visualized using 4′,6-diamidino-2-phenylindole (DAPI) (blue). Scale bars, 5 μm.

To further confirm the in vivo proteolytic processing of galectin-3 by meprin α/β heterodimers, immunoblotting of colonic tissue from wild-type, *mep1a^−/−^*, *mep1b^−/−^*, and *mep1a/b^−/−^* mice was performed (Fig. 2A and fig. S2). Notably, in tissue lysates of the entire colon of wild-type mice no full-length, only cleaved forms of galectin-3 with molecular masses of approximately 17 and 20 to 25 kDa could be detected. In contrast, in the *mep1a^−/−^*, *mep1b^−/−^*, and *mep1a/b^−/−^* mice, cleavage of galectin-3 was completely abolished, and only the full-length form of galectin-3 (~32 kDa) was visible. Hence, both meprin α and meprin β are required for the cleavage of galectin-3 in the colon under physiological conditions.

To investigate whether galectin-3 cleavage by the meprin α/β heterodimers takes place exclusively in the colon, we also examined whole tissue lysates of the kidney and the lung of wild-type,*mep1a^−/−^*, *mep1b^−/−^*, and *mep1a/b^−/−^* mice and as a control*lgals3^−/−^* mice (fig. S3, A and B). In the kidney of wild-type mice, where meprin α and meprin β are also known to be highly expressed, only the proteolytically processed and no full-length form of galectin-3 was detectable. Similar to the colon, galectin-3 cleavage was completely abrogated in the kidneys of *mep1a^−/−^*, *mep1b^−/−^*, and *mep1a/b^−/−^* mice (fig. S3A). In the lung, where the meprins are poorly expressed and not detectable via immunoblotting under physiological conditions, no galectin-3 processing was observed (fig. S3B). These results demonstrate that the cleavage of galectin-3 is organ-specific and depends on the expression of meprin α/β heterodimers.

As control experiment to assess whether the abolished galectin-3 cleavage in *mep1b^−/−^* mice can be rescued by reexpression of meprin β, we used Cre-inducible *Rosa26*^*mep1b*-*HA*^ mice. These Cre-inducible *Rosa26*^*mep1b*-*HA*^ mice contain a CAG promotor, a *loxP* site-flanked neomycin-Westphal stop cassette and the cDNA of hemagglutinin (HA)–tagged murine meprin β in the *Rosa26* locus (Fig. 2B). Crossing of *mep1b^−/−^;Rosa26*^*mep1b*-*HA*^ mice with villin-Cre transgenic mice leads to Cre-mediated recombination resulting in tissue-specific deletion of the stop cassette and expression of meprin β–HA in enterocytes. Consequently, the*mep1b^−/−^;Vil^Cre^*;*Rosa26^mep1b-HA^* mice have an overall meprin β knockout with reexpressed meprin β–HA in epithelial cells of the small and large intestines. In these mice, the galectin-3 cleavage could be completely rescued in the colon compared to *mep1b^−/−^* mice (Fig. 2B). Notably, we have previously shown that, compared to its homomeric form, meprin α is differentially glycosylated within the heterodimeric complex with meprin β, which explains the slight band shift for meprin α between the different genotypes (*7*). In addition, N-terminomics analysis of colonic tissue from *mep1b^−/−^;Vil^Cre^*;*Rosa26^mep1b-HA^* mice revealed no alterations in abundance of the proteolytic fragments of galectin-3 compared with wild-type animals (fig. S3C).

Galectin-3 is known to be released by the cell through a nonclassical secretory pathway (*33*) but can also be localized in the nucleus and the cytoplasm. We thus wanted to determine where galectin-3 and meprin α/β heterodimers interact. We used stimulated emission depletion (STED) microscopy to explore this possibility and stained for colonic galectin-3 and meprin β of either*mep1b^−/−^;Vil^Cre^*;*Rosa26^mep1b-HA^* mice, where meprin β can bemore easily detected via the HA-tag, or *mep1a/b^−/−^* mice(Fig. 2C and fig. S4). The immunofluorescence staining of*mep1b^−/−^;Vil^Cre^*;*Rosa26^mep1b-HA^* mice revealed that both proteins, meprin β and galectin-3, are mainly localized on the apical cell surface of colonic enterocytes. It appears that in the presence of meprin α/β heterodimers (*mep1b^−/−^;Vil^Cre^*;*Rosa26^mep1b-HA^*), galectin-3 is attached to the very apical site of the enterocytes, whereas in the absence of meprins (*mep1a/b^−/−^* mice), galectin-3 is more spread over the entire villin-stained area. In sum, we could validate that the meprin α/β heterodimers are responsible for the constitutive proteolytic processing of galectin-3 in mouse colon and kidney, which likely occurs at the apical plasma membrane of epithelial cells.

### Endogenous cleavage of human galectin-3 by meprin metalloproteases in differentiated enterocyte-like cells

**Fig. 3.**
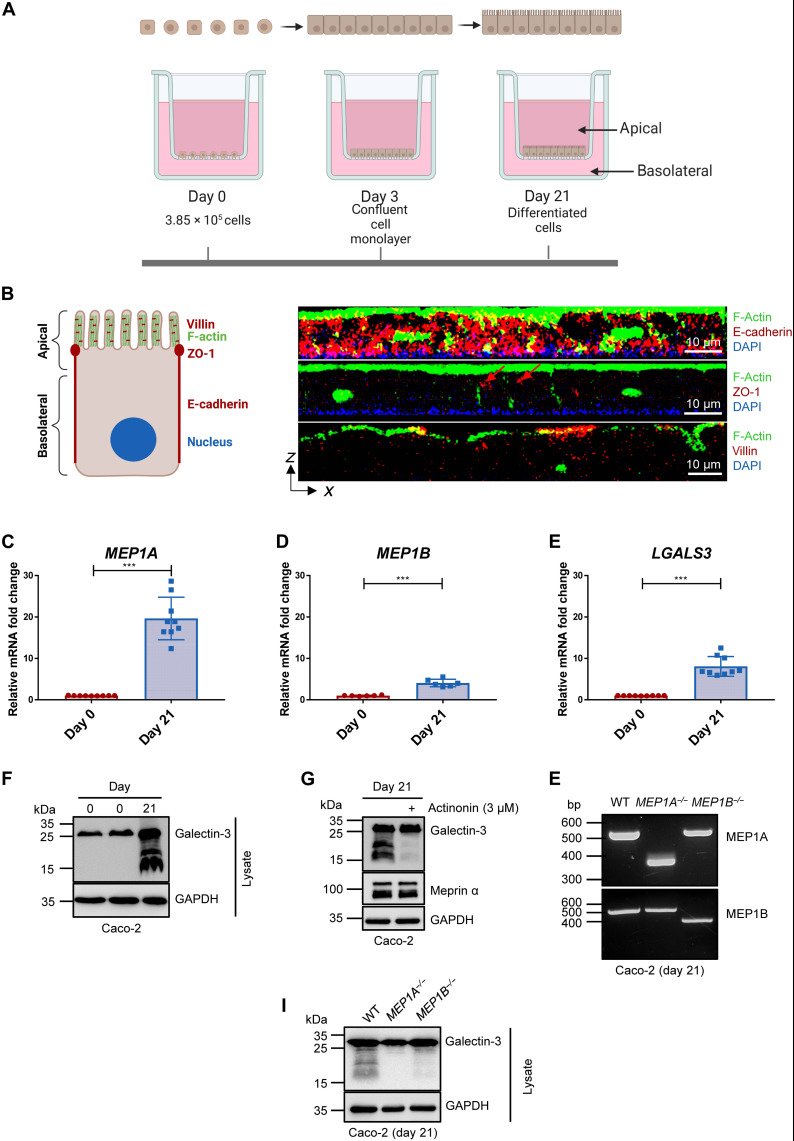
Endogenous galectin-3 cleavage by meprin metalloproteases in differentiated intestinal human cells. (**A**) Differentiation process of human colorectal adenocarcinoma Caco-2 cells seeded on a transwell for 21 days. (**B**) Scheme of an enterocyte (left) and cross-sectional view of the z-stacks from the cell monolayer of Caco-2 cells differentiated for 21 days (right) showing the localization of F-actin (green) and villin (red; bottom) on the apical side, ZO-1 (red; middle) between adjacent cells, and the nucleus (stained with DAPI; blue) on the basolateral side and E-cadherin (red; top) on the lateral membrane. (**C** to **E**) Relative mRNA expression of *MEP1A* (C), *MEP1B* (D), and *LGALS3* (E) in undifferentiated (day 0; red) and in differentiated (day 21; blue) Caco-2 cells (*n* = 9). Means ± SD. (***P < 0.001; unpaired *t* test). (**F**) Endogenous proteolytic processing of galectin-3 in cells of undifferentiated (day 0) and differentiated (day 21) Caco-2 was analyzed via Western blot analysis using a specific galectin-3 antibody. GAPDH served as a loading control. (**G**) Treatment of differentiated Caco-2 cells (day 21) with 3 μM actinonin showed an abolished galectin-3 cleavage analyzed by immunoblotting using a galectin-3–specific antibody. (**H**) Genotyping of the human meprin α and meprin β genes (*MEP1A* and *MEP1B*) in differentiated Caco-2 [wild-type (WT), *MEP1A^−/−^*, and *MEP1B^−/−^*] via polymerase chain reaction (PCR) using specific primers for validating CRISPR-Cas9 knockout of either meprin α or meprin β. (**I**) Western blot analysis investigating the cleavage of galectin-3 in cell lysates from differentiated (day 21) wild-type, *MEP1A^−/−^*, and *MEP1B^−/−^* Caco-2 cells generated with CRISPR-Cas9 genome editing.

To also examine the endogenous proteolytic processing of galectin-3 in a human system, we used a transwell approach with differentiated Caco-2 cells (Fig. 3A). Caco-2 is a human colorectal adenocarcinoma cell line that has the ability for spontaneous differentiation when reaching confluence (*34*). After differentiation, these cells develop morphogenic characteristics of absorptive enterocytes with an apical brush border (*35*). Morphological validation of Caco-2 cells differentiated for 21 days was performed by immunofluorescence staining and localization of F-actin, the nucleus, villin, zonula occludens-1 (ZO-1), and E-cadherin (Fig. 3B). Cross-sectional scanning of the z-stack of the entire cell layer showed F-actin and villin localized on the apical side, ZO-1 as a tight junction protein between adjacent cells and the nucleus on the basolateral side and E-cadherin on the transmembrane. The polarized and differentiated Caco-2 cells showed a significant increase in gene expression of *MEP1A*, *MEP1B*, and *LGALS3* in comparison to undifferentiated cells (Fig. 3, C to E). Western blot analysis revealed that only after differentiation, when the meprin expression is increased, galectin-3 is proteolytically processed endogenously (Fig. 3F). The galectin-3 cleavage could be inhibited by actinonin (Fig. 3G), a hydroxamate derivative, which is so far the most potent available inhibitor for both meprins (*K_i_* = 100 nM for meprin α and 2 μM for meprin β) (*36*).

**Fig. 4.**
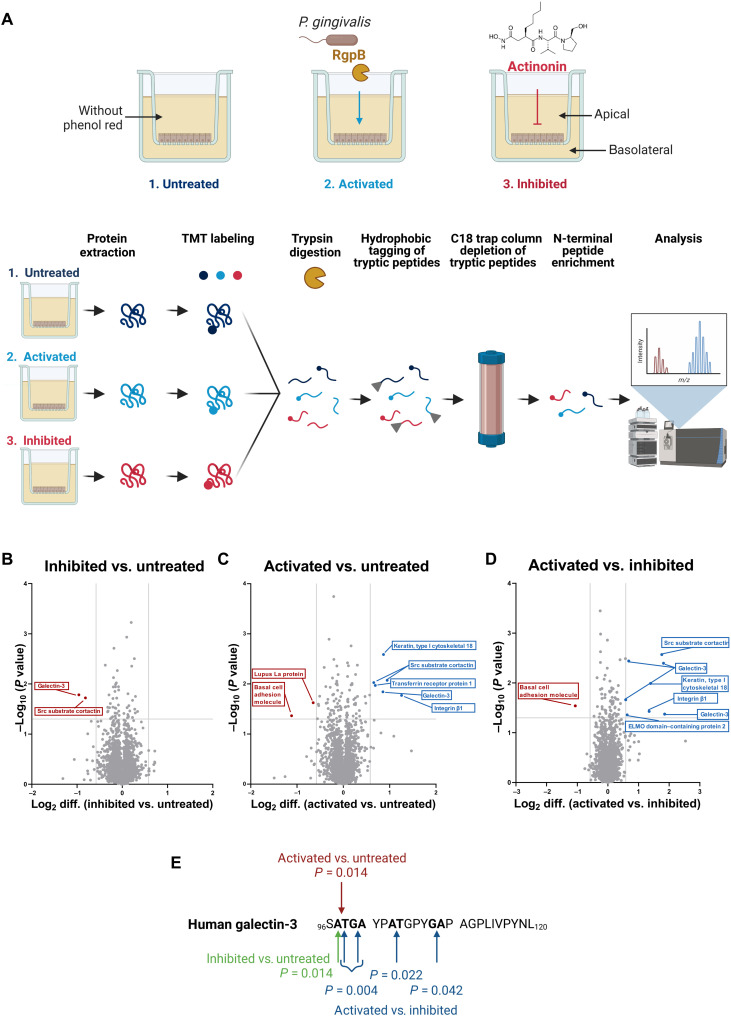
HYTANE analysis of differentiated intestinal human cells. (**A**) For Caco-2 cell treatment with activity modulating compounds, the medium was changed to serum-free Dulbecco’s modified Eagles’s medium (DMEM) without phenol red in both compartments on day 17. For the meprin inhibition, 3 μM actinonin was added daily to the apical and basolateral compartment until day 21 (red; right). For the meprin activation, 5 nM RgpB, a purified protease from *P. gingivalis*, was added to both compartments on day 20 (blue; middle). As a control, untreated cells were analyzed (left). Cells were harvested on day 21 for HYTANE analysis. (**B** to **D**) Volcano plots showing all identified proteolytic events in differentiated Caco-2 cells detected by HYTANE analysis (untreated, activated, and inhibited). Gray lines represent threshold values (±0.58 for log_2_ difference and *P* = 0.05). (**E**) Human galectin-3 sequence (amino acids 96 to 120) showing all identified cleavage sites using HYTANE analysis.

To further validate whether the endogenous cleavage of galectin-3 is induced by increased endogenous meprin expression during differentiation of Caco-2 cells, a clustered regularly interspaced short palindromic repeats–Cas (CRISPR-Cas) knockout of *MEP1A* (*MEP1A^−/−^*) or *MEP1B* (*MEP1B^−/−^*) was generated in these cells (Fig. 3H). After differentiation of Caco-2 *MEP1A^−/−^*, the galectin-3 cleavage was completely abrogated and almost fully abolished in Caco-2 *MEP1B^−/−^* cells (Fig. 3I). Notably, although the expression of meprins is markedly increased in differentiated Caco-2 cells, it is less pronounced compared to the in vivo situation, which likely explains the remaining full-length form of galectin-3 in our cell model. In addition to the knockout of the meprin genes and their inhibition by actinonin, we also applied meprin activators to see if we can increase the galectin-3 cleavage by stimulating meprin α and meprin β activity. Therefore, we performed N-terminomics analysis of Caco-2 cells that were treated with the bacterial protease arginine-gingipain B (RgpB) from the pathogen *Porphyromonas gingivalis*, which is well known to activate both meprin α and meprin β (*7*, *9*), and of Caco-2 cells treated with the inhibitor actinonin (Fig. 4A). We could validate galectin-3 as the main substrate for the meprin metalloproteases in this enterocyte-like human cell system. Cleavage of galectin-3 was more abundant in untreated than in inhibited cells, in activated than in untreated cells, and in activated than in inhibited cells (Fig. 4, B to D). The cleavage of galectin-3 by meprins was identified to occur within the N-terminal tail (Fig. 4E) close to the C-terminal CRD (amino acids 132 to 264), which is in line with our observations from mouse colonic tissues (Fig. 1B). Together, we demonstrated that murine and human galectin-3 is constitutively cleaved by meprin α/β heterodimers in enterocytes, which can be inhibited or induced by meprin activity modulating compounds.

### Meprin β strongly interacts with full-length and cleaved galectin-3

**Fig. 5.**
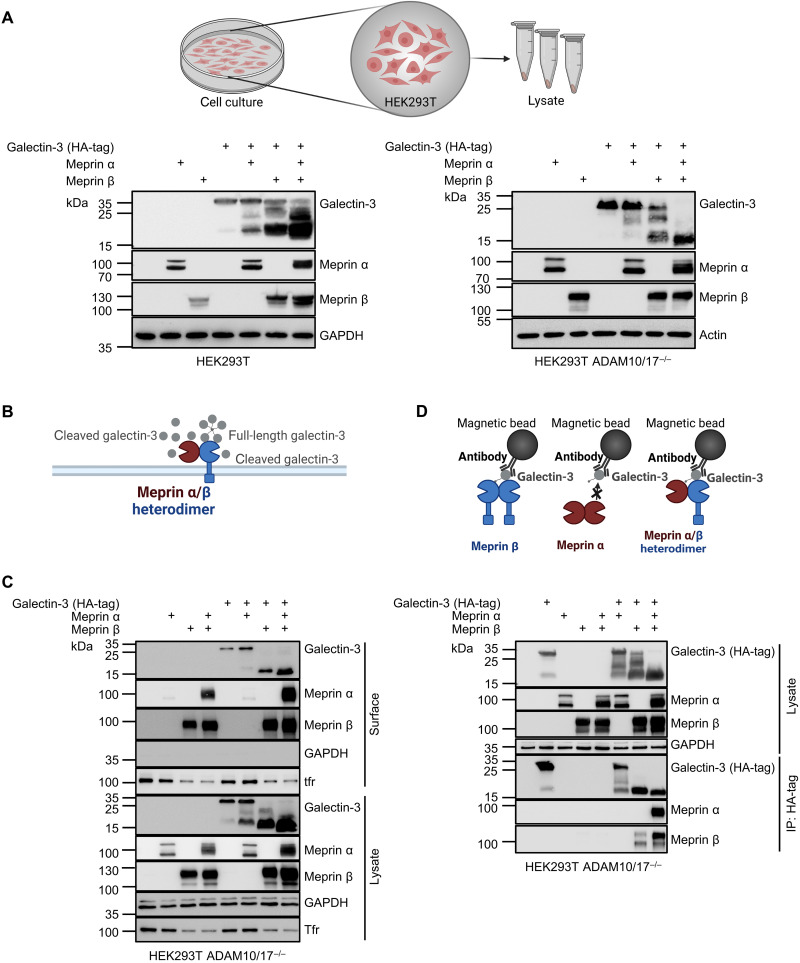
Analyzing biochemical properties of proteolytic cleavage and interaction of galectin-3 with meprin metalloproteases using transiently transfected cells. (**A**) Detection of galectin-3 cleavage fragments by Western blot analysis upon coexpression of galectin-3 (C-terminally HA-tagged) with meprin α and/or meprin β in wild-type HEK293T cells and HEK293T-deficient for ADAM10 and ADAM17 (ADAM10/17^−/−^). (**B** and **C**) Cell surface biotinylation assay of HEK293T ADAM10/17^−/−^ cells transfected with galectin-3, meprin α, and/or meprin β (C). Cell surface proteins were labeled with primary amine biotinylation, pulled down with magnetic streptavidin beads, and analyzed via Western blot analysis. Transferrin receptor (Tfr) and GAPDH served as a control. Cartoon of the proteolytic processing of galectin-3 by meprin α/β heterodimers at the membrane (B). (**D**) HEK293T ADAM10/17^−/−^ cells were transfected with galectin-3 and meprin α and/or meprin β. After cell lysis, co-IP was performed using an HA-tag antibody against galectin-3 C terminus. Lysate controls and immunoprecipitates were analyzed by Western blot.

To investigate the biochemical properties of galectin-3 cleavage by meprin α/β heterodimers in more detail, we used the easy to manipulate human cell line human embryonic kidney (HEK) 293T. First, we validated the cleavage of human galectin-3 by human meprin α/β heterodimers upon transient expression of these proteins in HEK293T cells. In addition, we used HEK293T cells that were deficient for the proteases ADAM10 and ADAM17 (HEK ADAM10/17^−/−^) preventing meprin β shedding (*16*–*19*) to see if the interaction of galectin-3 and the meprin α/β heterodimer takes place predominantly at the cell surface (Fig. 5A). Cotransfection of galectin-3 with meprin α or meprin β demonstrated that both proteases are able to cleave galectin-3, but only the cotransfection of galectin-3 with both meprin α and meprin β resulted in the full conversion of the full-length galectin-3 into a remaining 17-kDa fragment. In addition, the cleavage was more efficient in cells with inhibited meprin β shedding (Fig. 5, A and B). Notably, to get an efficient proteolytic processing of the full-length galectin-3, enzymatic activity of both meprins is required, which we could demonstrate with the use of catalytically inactive variants of meprin α and meprin β (fig. S5A).

By using a protein-biotinylation assay, biotinylated full-length and the cleaved form of galectin-3 could be identified suggesting their presence at the cell surface (Fig. 5C). Coimmunoprecipitation (co-IP) experiments revealed that nonproteolytic interaction with galectin-3 only takes place via meprin β but not via meprin α. In the meprin α/β heterodimer complex, meprin α and galectin-3 only come into close proximity through their common binding partner meprin β (Fig. 5D). This conclusion was validated using a catalytically inactive variant of meprin β (meprin β–E153A), demonstrating that meprin β can interact with both the full-length and with the cleaved form of galectin-3 (fig. S5B).

### Only membrane-bound and not soluble meprin β can cleave galectin-3

**Fig. 6.**
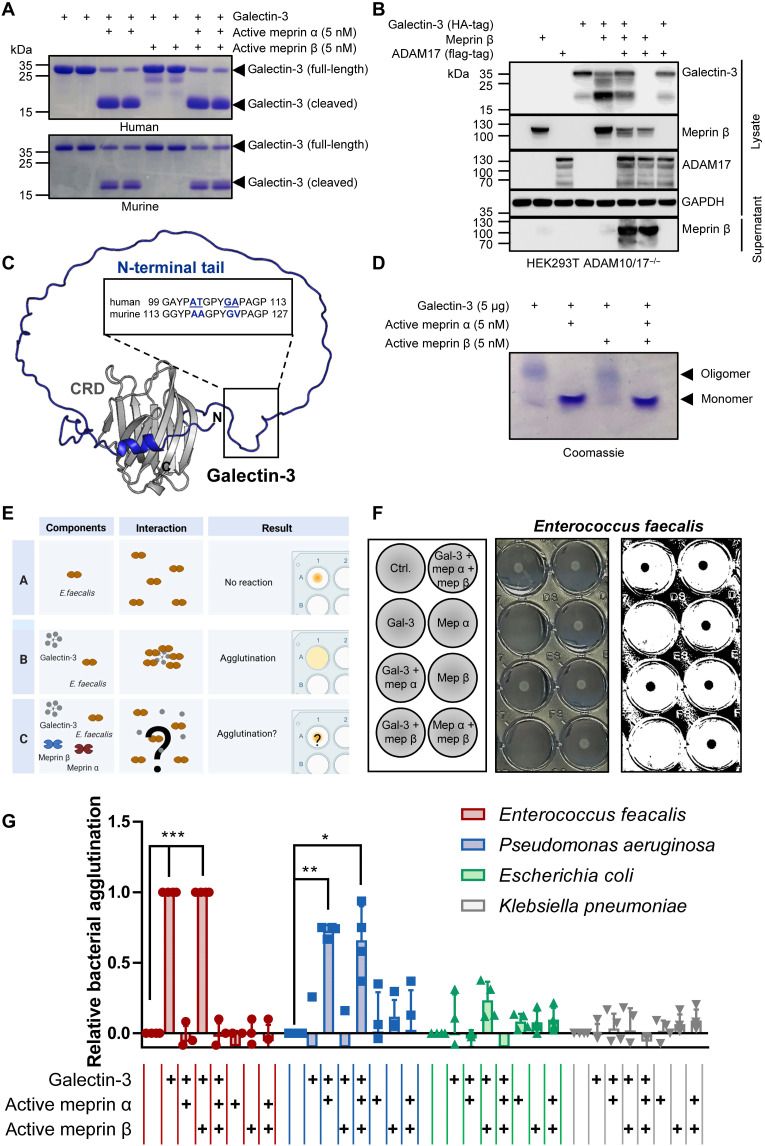
Cleavage of galectin-3 alters its oligomeric structure and bacterial agglutination properties. (**A**) Recombinant human (top) and murine (bottom) galectin-3 was incubated with recombinant active meprin α and/or meprin β and analyzed by SDS-PAGE and Coomassie brilliant blue staining. (**B**) Galectin-3 cleavage analyzed in transfected HEK293T ADAM10/17^-/-^ cells in the presence or absence of the meprin β sheddase ADAM17 detected by immunoblotting. (**C**) The cleavage site in human and murine galectin-3 was identified using LC-MS analysis of the gel bands in (A) as depicted in the structural model of galectin-3 (AlphaFold: AF-P16110-F1). Gray, CRD; blue, N-terminal tail. (**D**) Analysis of human recombinant galectin-3 with and without cleavage by meprins via native PAGE. (**E**) Scheme of the bacterial agglutination assay. (**F**) *E. faecalis* was incubated with recombinant galectin-3 alone or preincubated with active recombinant meprin α and/or meprin β, and bacterial agglutination was examined. As a negative control buffer, 20 mM Hepes, pH 7.2, or active recombinant meprins were applied without galectin-3. Middle panel original image. Right panel shows the area of nonagglutinated bacteria, which was calculated using ImageJ Fiji. All values were normalized to the control (Hepes), while the control corresponds to 0 and maximum agglutination to 1. (**G**) Relative bacterial agglutination of *E. faecalis* (*n* = 4), *P. aeruginosa* (*n* = 4), *E. coli* (*n* = 4), and *K. pneumoniae* (*n* = 4) incubated with galectin-3 or galectin-3 preincubated with meprin α and/or meprin β. Data are presented as means ± SD, and statistical analysis was assessed by two-way analysis of variance (ANOVA), followed by a Tukey posttest (*P < 0.05; **P < 0.01; ***P < 0.001).

To validate the exact meprin cleavage sites in galectin-3, human and murine recombinant galectin-3 was incubated with active recombinant meprin α and/or meprin β lacking its transmembrane domain. Subsequently, galectin-3 proteolytic fragments were separated via SDS–polyacrylamide gel electrophoresis (PAGE) and visualized by Coomassie brilliant blue staining (Fig. 6A). Notably, we barely detected galectin-3 cleavage fragments in the presence of soluble active recombinant meprin β. Therefore, we hypothesized that membrane-tethering of the protease might be a prerequisite for the physiological processing of galectin-3, which is in line with the biotinylation and IP experiments using the HEK293T cells deficient for ADAM10 and ADAM17, thus preventing meprin β shedding, as well as with STED microscopy. To investigate whether soluble meprin β is unable to cleave galectin-3 in a cellular environment, we analyzed transfected HEK293T ADAM10/17^−/−^ cells. Coexpression of meprin β and its sheddase ADAM17 resulted in diminished cleavage of galectin-3 (Fig. 6B).

In contrast, soluble recombinant meprin α efficiently cleaved galectin-3, which could not be inhibited by lactose (fig. S5C). The resulting fragments (Fig. 6A) were excised and analyzed by liquid chromatography-MS (LC-MS). Analysis of the obtained fragments confirmed that meprin α cleaves murine galectin-3 most prominently at position Gly^122^/Val^123^. For human galectin-3, we detected cleavage between Gly^108^ and Ala^109^, which corresponds to the same position in murine galectin-3 based on a sequence alignment (Fig. 6C). Another less prominent cleavage site identified in human galectin-3 between Ala^103^ and Thr^104^ was also conserved in murine galectin-3 between Ala^117^ and Ala^118^. Hence, the cleavage of galectin-3 by meprin α occurs very close to the CRD and results in the loss of the N-terminal part of galectin-3. On the basis of the fact that galectin-3 is able to form pentamers via its N-terminal tail (*37*–*40*) and that this molecular assembly seems to be proteolytically regulated (*37*, *41*–*45*), we performed a native PAGE to visualize potential galectin-3 oligomerization. Here, we could show that the cleavage of galectin-3 by recombinant meprin α clearly prevents oligomerization of galectin-3 (Fig. 6D). Hence, this oligomerization of galectin-3 is disrupted by the degradation of its N-terminal part by the metalloprotease meprin α and, thus, by the heterodimer.

In summary, the cleavage site in galectin-3 could be identified and is structurally conserved in human and mouse protein. The proteolytic processing of galectin-3 regulates its oligomerization via its N-terminal tail.

### Proteolytic processing of galectin-3 leads to altered bacterial agglutination

It has been reported that cleavage of galectin-3 by MMP2 and MMP9 in its N-terminal tail abolishes its capacity for hemagglutination and self-association (*44*). Moreover, it was shown that galectin-3 is able to bind and agglutinate certain bacteria (*24*–*27*). Therefore, we performed a bacterial agglutination assay to study whether cleavage of galectin-3 by meprins has an impact on its ability to agglutinate the bacteria *Enterococcus faecalis*, *Pseudomonas aeruginosa*, *Escherichia coli*, and *Klebsiella pneumoniae*. In case of agglutination, network-like structures are formed, which means that the bacteria are no longer visible because of a diffuse distribution all over the well (Fig. 6, E and F). We preincubated recombinant galectin-3 with and without recombinant meprin α and/or meprin β to obtain full-length and cleaved galectin-3. While these different conditions did not induce agglutination upon incubation with *E. coli* and *K. pneumoniae* (Fig. 6G and fig. S6, A and B), full-length galectin-3 profoundly agglutinated *E. faecalis* (Fig. 6, F and G, and fig. S6C). Notably, this agglutination was completely abrogated when galectin-3 was cleaved by meprin α alone or meprin α and meprin β prior administration to the bacteria. For *P. aeruginosa* incubated with full-length galectin-3, no agglutination could be observed. However, galectin-3 cleaved by meprin α alone or by meprin α and meprin β showed high agglutination of *P. aeruginosa* (Fig. 6G and fig. S6D).

Here, we demonstrated that proteolytic processing of galectin-3 by meprin α can regulate bacterial agglutination. Notably, only certain bacterial species were able to agglutinate either with cleaved or full-length galectin-3. Hence, we hypothesized that proteolysis of galectin-3 in vivo may be involved in defining the microbiome colonization of the intestine.

### Loss of galectin-3 revealed alterations in microbiome but not mucus properties in mouse colon

**Fig. 7.**
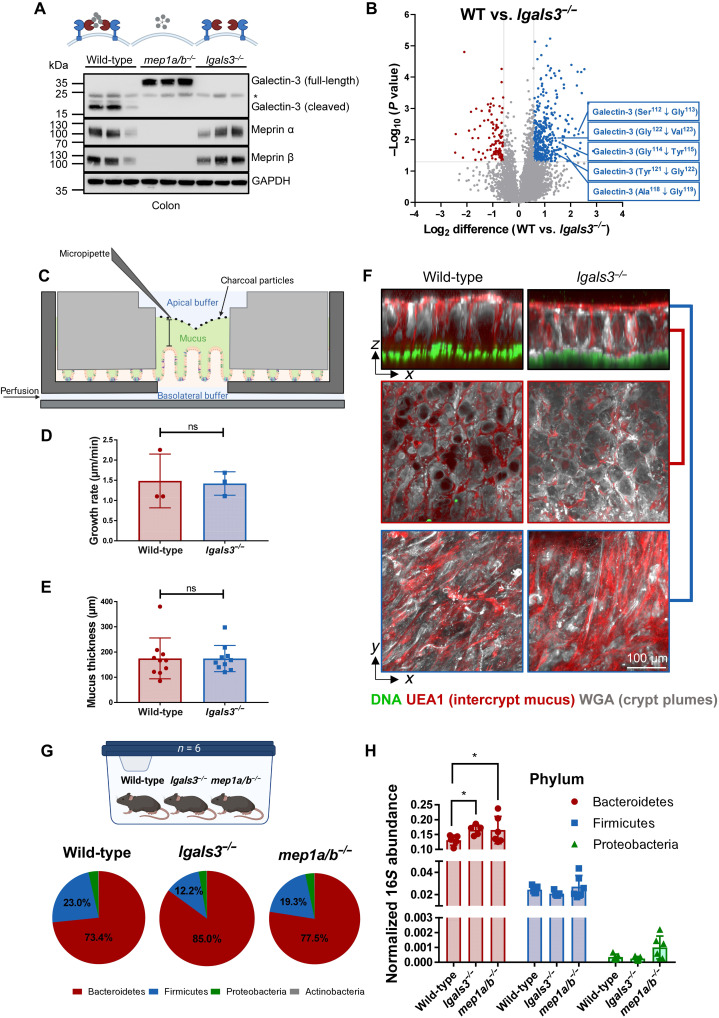
Loss of galectin-3 revealed alterations in microbiome composition but not in the mucus properties. (**A**) Immunoblot analysis of colonic lysates from wild-type, *mep1a/b^−/−^*, and *lgals3^−/−^* mice. Asterisk marks an unspecific band. (**B**) Volcano plot showing N termini identified by HYTANE analysis of wild-type versus *lgals3^−/−^* mice. Gray lines represent threshold values (±0.58 for log_2_ difference and *P* = 0.05). (**C**) Scheme of a horizontal perfusion chamber for mucus growth rate analysis. (**D**) Mucus thickness was measured at 0, 15, and 30 min after mounting, and growth rate was calculated as the delta mucus thickness divided by time from wild-type and *lgals3^−/−^* mice (*n* = 3). (**E**) The mucus thickness was quantified in wild-type and *lgals3^−/−^* mice by calculating the average tissue to beads distance in the penetrability assay (*n* = 10). (D and E) Data are represented as means ± SD, and statistical analysis was assessed by an unpaired *t* test [ns (not significant)]. (**F**) Cross sections through the inner mucus layer of ex vivo wild-type and *lgals3^−/−^* distal colon tissue as well as *y*/*x*-axis images in the inner mucus layer (red line) and on the mucus surface (blue line): DNA (green), *U. Europaeus* agglutinin I (UEA-1)–stained intercrypt mucus (red), wheat germ agglutinin (WGA)–stained (gray). Scale bar, 100 μm. (**G**) Shallow shotgun sequencing of the microbiome of wild-type, *lgals3^−/−^*, and *mep1a/b^−/−^* from stool samples (*n* = 6). (**H**) Normalized 16*S* abundance analyzed by qRT-PCR from stool samples of *lgals3^−/−^* and *mep1a/b^−/−^* mice in comparison to wild-type mice. Data are represented as means ± SD, and statistical analysis was assessed by two-way ANOVA, followed by a Tukey posttest (*P < 0.05).

To examine the physiological role of the proteolytic processing of galectin-3 by the meprin α/β heterodimers, we investigated the colon of mice deficient for galectin-3 (*lgals3^−/−^*). Immunoblot analysis revealed no alterations in protein expression or band pattern of meprin α and meprin β in the *lgals3^−/−^* mice in comparison to wild-type mice (Fig. 7A). Furthermore, N-terminomics analysis of wild-type versus *lgals3^−/−^* mice demonstrated that under physiological conditions, galectin-3 is preferably cleaved between Ser^112^ and Gly^113^ (Fig. 7B) at the exact major cleavage site that we identified for meprins. This further corroborated that the meprin α/β heterodimer complex is the main protease responsible for the proteolytic processing of galectin-3 within the colon. As galectin-3 has been reported to alter MUC2 expression and interact with MUC2 directly (*23*, *46*), we investigated whether loss of galectin-3 had an impact on the mucus properties in the colon. Colon explants were mounted in a perfusion chamber (Fig. 7C) as described before, and the surface of the mucus was visualized by charcoal or fluorescent beads (*47*). Mucus growth, thickness, structure, or penetrability was not altered in *lgals3^−/−^* mice compared to wild-type mice (Fig. 7, C to F, and fig. S7, A and B).

The observation that galectin-3 did not affect mucus properties but could agglutinate bacteria prompted us to analyze the fecal microbiota. Wild-type, *lgals3^−/−^*, and *mep1a/b^−/−^* mice were cohoused in the same cages for more than 6 weeks, and their feces microbiome was analyzed with the use of shallow shotgun next-generation sequencing. The *lgals3^−/−^* mice revealed increased relative abundance of Bacteroidetes and decreased abundance of Firmicutes (Fig. 7G) compared to cohoused wild-type mice. These changes in the microbiota were in line with alterations observed for the *mep1a/b^−/−^* mice, maybe as a consequence of lack of the proteolytic processing of galectin-3 by meprin α/β heterodimers. Quantitative analysis of the relative abundance of 16*S* ribosomal RNA (rRNA) from the phyla Bacteroidetes, Firmicutes, and Proteobacteria in stool by quantitative real-time polymerase chain reaction (qRT-PCR) showed significantly increased Bacteroidetes in both *lgals3^−/−^* and *mep1a/b^−/−^* mice (Fig. 7H). Notably, we additionally observed a high number of proteins that were equally regulated by both the *mep1a/b* and *lgals3* genes (figs. S8, A and B, and S9, A to C), which implicates a functional interaction of the meprin–galectin-3-complex that goes beyond microbial homeostasis. A biochemical explanation might be that galectin-3 is able to increase meprin β activity on the cell surface (fig. S8, C and D) probably by stabilizing meprin β at the plasma membrane as it was shown for vascular endothelial growth factor receptor 2 (*48*).

### The bacterial load and composition regulate galectin-3 cleavage by meprin metalloproteases in vivo

**Fig. 8.**
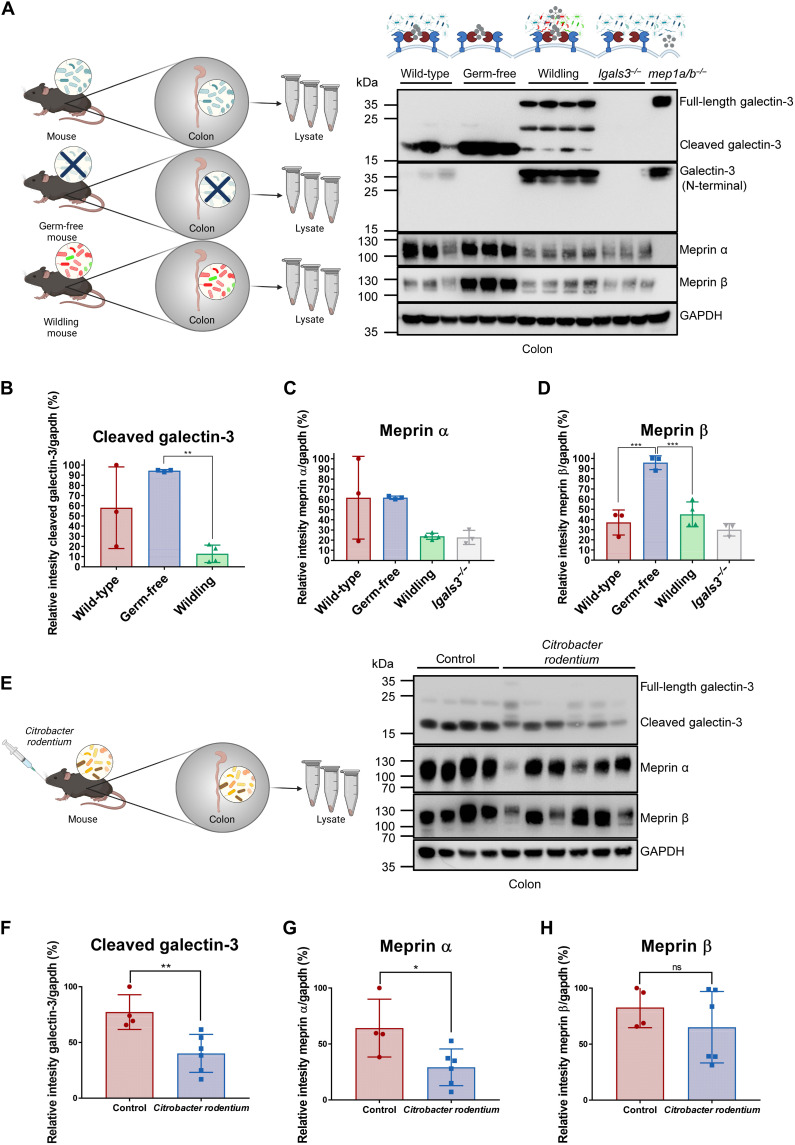
The bacterial load and composition regulate galectin-3 cleavage by meprin metalloproteases in vivo. (**A**) Western blot analysis of colon tissue from conventionally housed (wild-type), germ-free, or wildling mice demonstrated an altered cleavage of galectin-3 in response to different microbiomes. (**B** to **D**) Densitometric analysis of cleaved galectin-3 (B), meprin α (C), and meprin β (D) calculated by ImageJ from three biological replicates of wild-type and germ-free mice and four biological replicates of wildling mice as shown in (A). Data are represented as means ± SD, and statistical analysis was assessed by one-way ANOVA, followed by a Tukey posttest (**P < 0.01; ***P < 0.001). (**E**) Analysis of proteolytic processing of galectin-3 and meprin expression in the colon of *C. rodentium*–infected mice (control, *n* = 4; *C. rodentium*, *n* = 6) 7 days after infection via immunoblotting. (**F** to **H**) Densitometric analysis of cleaved galectin-3 (F), meprin α (G), and meprin β (H) calculated by ImageJ from four or six biological replicates as shown in (E). Data are represented as means ± SD, and statistical analysis was assessed by an unpaired *t* test (ns, *P* > 0.05; *P < 0.05; **P < 0.01). (B to D and F to H) The intensity of cleaved galectin-3, meprin α, and meprin β was determined relative to the normalized expression of GAPDH.

We next wanted to assess the potential microbial regulation of galectin-3 processing by the meprin α/β heterodimers and its physiological importance for host-microbiome interaction. To observe whether galectin-3 cleavage and meprin expression is altered in response to different bacterial loads, immunoblotting of colon tissue from germ-free, conventionally housed (specific pathogen-free), or wildling mice revealed a profound regulatory effect of the microbiome on galectin-3 cleavage and meprin expression (Fig. 8, A to D). Germ-free mice showed a significant accumulation of membrane-bound meprin β (Fig. 8, A and D), which is in line with previous observations indicating that bacterial factors stimulate the shedding of meprin β (*8*, *9*). Analyzing colon tissue from wildling mice, which contain a more physiological microbiome than conventionally raised mice (*49*) revealed enhanced shedding of meprin β and consequently significant less cleaved galectin-3 (Fig. 8, A and B). Thus, the cleavage of galectin-3 by meprin α/β heterodimers seems to be strongly regulated and dependent on the intestinal microbiome composition.

It is well known that a complex intestinal microbiota is not only beneficial for the host with regard to metabolism and immunity but is also well accepted that the dysregulated microbiome has a key impact on IBD (*50*, *51*). Therefore, we were interested in the proteolytic processing of galectin-3 by meprin α/β heterodimers upon infection and the subsequent inflammation (Fig. 8E). In mice infected with *Citrobacter rodentium*, the levels of cleaved galectin-3 and meprin α were significantly down-regulated in comparison to mock-infected mice (Fig. 8, E to H).

Decreased levels of galectin-3, meprin α, and meprin β have been observed to correlate with the severity of inflammation in patients with IBD (*6*, *29*, *31*, *52*, *53*). In human colonic biopsies from patients with ulcerative colitis (UC), full-length galectin-3 was significantly increased and cleaved galectin-3 was consequently decreased in comparison to the healthy controls (fig. S10, A and B).

Hence, the proteolytic processing of galectin-3 by meprin α/β heterodimers contributes to the host-microbiome interaction in health and disease. Our study demonstrates that the gut microbiome regulates the localization and activity of the host-enzyme-complex meprin α/β, which in turn regulates the bacterial agglutination properties of galectin-3.

## DISCUSSION

In this study, we could show that constitutive proteolytic processing of galectin-3 by meprin α/β heterodimers in the colon seems to be an important mediator of bacterial agglutination and the microbiome homeostasis that could contribute to the protection of the host epithelium against potential pathogens. The mucosal integrity and the bacterial homeostasis of the entire intestinal tract are crucial for health and disease. Dysregulation is associated with infection and inflammation such as IBD. Alterations in mucus properties, attachment, or host-microbiome interaction are important hallmarks of IBD including Crohn’s disease (CD) and UC (*3*, *54*, *55*). Decreased levels of meprin α and meprin β correlate with the severity of inflammation in patients with IBD (*6*, *52*). Furthermore, *mep1a/b^−/−^* mice suffer from a more severe disease progression in DSS-induced colitis than wild-type mice (*5*). Therefore, meprin α and meprin β seem to be important for the gut homeostasis.

We demonstrated previously that meprin β is responsible for mucus detachment in the small intestine and therefore protects the host epithelium against bacterial overgrowth and infection (*8*). Usually, the mucus layer is loosely attached to the host epithelium in the small intestine, leading to mucus detachment and fast mucus renewal important for intestinal homeostasis. In contrast, in *mep1b^−/−^* mice, the small intestinal mucus is densely packed and tightly attached to the epithelial surface (*8*). This mucus homeostasis is microbiome dependent because germ-free mice also exhibit mucus attached to the host small intestine epithelium (*8*). Thus, meprins play a crucial role in mucosal integrity, which in turn is associated with and regulated by the microbiome. Several studies suggested that bacteria and the microbiome play an important role in the onset and duration of IBD (*54*). In human ileal lesions of patients with CD, meprins act as a host defense mechanism to counteract bacterial colonization of pathogenic adherent-invasive *E. coli* by proteolytic degradation of type 1 pili preventing bacterial adhesion or invasion to the intestinal epithelial cells (*56*). Our observations indicate that both meprin α and meprin β play an important role for the gut homeostasis by regulating mucosal integrity and bacterial composition and that a balance of these proteases might play a role in the progression of IBD.

When meprin α and meprin β are coexpressed, particularly in the colon, they form a covalently-linked heterodimeric complex in the early endoplasmic reticulum, which is transported to the cell surface, retaining meprin α on the plasma membrane (*7*). However, the functional consequence and the intestinal substrates for the meprin α/β heterodimer have remained elusive. Therefore, we investigated the physiological role of the meprin metalloproteases and especially the function of meprin α/β heterodimers. A knockout of both proteases (*mep1a/b^−/−^*) in mice causes a significant alteration in the microbiome composition in cohoused animals, illustrating the importance of both proteases for healthy gut homeostasis. By applying MS-based N-terminomics, we identified galectin-3 as the major intestinal substrate for meprin α/β heterodimers regulating bacterial agglutination and composition in the gut.

Although several other cleavage events in galectin-3 have been described before (see annotations in the TopFIND database; https://topfind.clip.msl.ubc.ca) (*57*), none of these were observed in our N-terminomics analyses. The former studies are all based on in vitro assays with purified proteins and cultured cell lines, which underlines the necessity to perform unbiased substrate screenings from biological samples.

Within the gut galectin-3 is predominantly expressed in the villus tips (*22*) and is also described to interact with MUC2 (*23*), which is the known meprin β substrate in the small intestine (*8*). Notably, *mep1a*, *mep1b*, and *lgals3* are suggested as marker genes for enterocytes (*4*). Galectin-3 has multiple suggested biological functions depending on its localization (*58*) and may play important roles in several diseases (*59*). However, the physiological function of intestinal galectin-3 in vivo remains unknown. Galectin-3 is a susceptibility gene for IBD, and mice deficient for galectin-3 (*lgals3^−/−^*) suffer from a more severe disease progression in DSS-induced colitis model in comparison to wild-type mice (*31*), which is in line with *meb1a/b^−/−^* animals. Moreover, the galectin-3 levels in serum from patients suffering from UC and CD were significantly elevated (*28*).

Galectin-3 is known to interact with commensal and pathogenic microorganisms (*24*, *26*, *27*, *60*, *61*), suggesting a function of galectin-3 in bacterial homeostasis. Previously, it was shown that galectin-3 is able to trap and aggregate bacteria, which is an important host protective mechanism of bacterial infection and colonization (*27*). For example, during experimental *Helicobacter pylori* infection, bacterial cells were mostly trapped on the surface mucus layer in wild-type mice, whereas the bacteria infiltrated deep into the gastric glands in galectin-3–deficient mice. In our study, we could show that galectin-3 is able to agglutinate *E. faecalis* and *P. aeruginosa.* This trapping was further regulated by the proteolytic processing of galectin-3 by meprins. *E. faecalis* was agglutinated by full-length galectin-3, which was abolished after meprin cleavage, whereas *P. aeruginosa* agglutinates only with the cleaved form of galectin-3. Intact galectin-3 is able to oligomerize via its N-terminal domain and thereby cross-link and agglutinate bacteria. However, how galectin-3 can be exploited by pathogens is more difficult to understand. The pathogen *P. aeruginosa* was not agglutinated by intact galectin-3 but well by the cleaved form. Previous studies on galectin-3 have suggested bacterial agglutination and binding of the bacteria to epithelial cells (*24*), and others demonstrated that galectin-3 oligomerization can be regulated by certain proteolytic enzymes, such as bacterial collagenase IV, MMP2, and MMP9 (*37*, *38*, *42*–*44*). These enzymes are able to cleave galectin-3 within its linker region, which results in the release of the N-terminal tail that is responsible for pentameric oligomerization leading to loss of hemagglutination and self-association. As described, an oligomerization of galectin-3 is possible either by interactions of the N-terminal tails, via the CRDs, or by an interplay of both the CRD and the N-terminal tail (*62*, *63*). A so-called C-type dimerization/trimerization of the CRDs alone has been shown using electron microscopy and additionally in solution by fluorescence anisotropy (*62*, *64*). The exact mode of carbohydrate domain-dimer formation is currently not fully understood. However, we speculate that in case of galectin-3 cleavage by meprin α, the oligomerization state shifts from the preferred pentameric structure stabilized via N-terminal interactions to CRD-facilitated dimers/trimers. Consequently, the availability of ligands and/or carbohydrates of the different bacteria may lead to the different agglutination effects of full-length and meprin α–cleaved galectin-3, as observed in our study for *E. faecalis* in comparison to *P. aeruginosa*.

We now demonstrate that in the colon and kidney, galectin-3 is constitutively cleaved in vivo by meprin α/β heterodimers, which is able to regulate bacterial agglutination and microbial homeostasis in the gut, as demonstrated in *lgals3^−/−^* and *mep1a/b^−/−^* mice. Furthermore, we could show that the proteolytic processing of galectin-3 by meprin α/β heterodimers is regulated by the microbiome. The complete lack of bacteria in germ-free mice revealed increased level in cleavage of galectin-3, whereas a microbiome typical for mice living in the wild diminished galectin-3 processing pinpoint to highly adaptable molecular system that is modulated by bacterial factors.

In conclusion, our results indicate that the proteolytic processing of galectin-3 by meprin α/β heterodimers contributes to the mucosal barrier properties that protect the host epithelium against commensal and pathogenic bacteria by modulating the interaction between microbes and the host mucosa and the bacterial composition.

## MATERIALS AND METHODS

### Chemicals

All chemicals were of analytical grade and obtained from Carl Roth (Karlsruhe, Germany), Gibco (Waltham, MA, USA), Merck (Darmstadt, Germany), MilliporeSigma (Burlington, MA, USA), or Thermo Fisher Scientific (Waltham, MA, USA), if not stated otherwise. Primers were synthesized by Merck (Darmstadt, Germany).

### Animals

All mice were on a C57BL/6 background and maintained under constant environmental conditions (12-hour light-dark cycles, temperature) and in an individually ventilated cage system with food and drinking water ad libitum in accordance with the ethical standards and current guidelines of the European Union. All animal protocols were approved by the Central Animal Facility of the University of Kiel and the National Animal Care Committee of Germany as well as by the Institutional Review Board of the Medical University of Vienna and the Austrian Ministry of Sciences. Mice were used between 8 and 40 weeks of age. Both sexes of mice were used equally throughout the study.

### Generation of mouse lines

The generation of *mep1b^−/−^* and *mep1a^−/−^* mice was previously described (*6*, *65*). Mice homozygote deficient for meprin α and meprin β (*mep1a/b^−/−^*) were received by crossing *mep1a^−/−^* and *mep1b^−/−^* mice. *Lgals3^−/−^* mice were obtained from the Jackson Laboratories (*B6.Cg-Lgals3^tm1Poi^/J*, #006338).

#### Generation of meprin β knock-in mice

Meprin β knock-in mice were generated in collaboration with R. Naumann and M. Haase from the Max Planck Institute of Molecular Cell Biology and Genetics in Dresden as described before (*66*). In summary, a cDNA coding for C-terminally HA-tagged murine meprin β was cloned into a CAG-Cre-IRES-*Rosa26* vector. With the use of this vector, the cDNA was inserted into the *Rosa26* locus of C57BL/6 N mice by homologous recombination. Homozygous meprin β knock-in mice are referred to as *Rosa26*^*mep1b*-*HA*^. A floxed neomycin-Westphal stop cassette between a CAG promotor and the meprin β cDNA was prevented from transcription meprin β cDNA until it is excised by a Cre recombinase (*66*). To achieve meprin β overexpression only and specific in the gut, *Rosa26*^*mep1b*-*HA*^ mice were crossed with *mep1b^−/−^* mice (*65*) and mice expressing Cre recombinase under the control of a 9-kb regulatory region of the murine villin gene (*Vil^Cre^*), specifically active in the entire intestinal epithelium (*67*). *Rosa26*^*mep1b*-*HA*^ mice, which were homozygous for *mep1b^−/−^* and heterozygous for *Vil^Cre^*, are termed as *mep1b^−/−^;Vil^Cre^*;*Rosa26^mep1b-HA^* mice.

### Tissue collection and lysis

Mouse tissue from *mep1a^−/−^*, *mep1b^−/−^*, *mep1a/b^−/−^*, *lgals3^−/−^*, and *mep1b^−/−^;Vil^Cre^*;*Rosa26^mep1b-HA^* mice was isolated from mice euthanized with cervical dislocation according to the Guide for the Care and Use of Laboratory Animals (German Animal Welfare Act on Protection of Animals). The tissues from germ-free mice (*n* = 3) were provided to us by J. F. Baines (Max Planck Institute for Evolutionary Biology, Plön) and S. M. Ibrahim (Institute of Experimental Dermatology, Lübeck). The tissue of wildling animals (*49*) (*n* = 4) were received from the mouse facilities at the Medical Center–University of Freiburg, Germany. All the tissues were homogenized in lysis buffer [1× cOmplete protease inhibitor cocktail (Roche) and 1% (v/v) Triton X-100 in phosphate-buffered saline (PBS) (pH 7.4)] using the Precellys 24 (VWR) for 3 cycles at 6500 rpm and 4°C. The homogenates were incubated for 2 hours at 4°C and, afterward, centrifuged for 30 min at 13,500*g* at 4°C. The protein concentration of all lysates was determined using the Pierce BCA (bicinchoninic acid) Protein Assay Kit (Thermo Fisher Scientific) according to the manufacturer’s instructions.

### HYTANE analysis

Colon tissues (*n* = 3) excluding the caecum and Caco-2 lysates were sonicated and centrifuged at 20,000*g* for 5 min at 4°C, and each sample was methanol/chloroform/water (MCW)–precipitated to remove any interfering substances such as small primary amines. Samples were resuspended in 6 M guanidine-HCl in 100 mM triethylammonium bicarbonate (TEAB), and the BCA Protein Assay on a diluted fraction was performed to determine protein concentration.

Approximately 100 μg of each sample was reduced with tris(2-carboxyethyl)phosphine (TCEP; 5 mM final concentration) for 30 min at 65°C and then alkylated with iodoacetamide (12.5 mM final concentration) at room temperature for 15 min. Samples were then labeled with tandem mass tag (TMT) reagent (TMT-6-plex) in equal volume of dimethyl sulfoxide (DMSO) so that the final concentration of DMSO was 50%. The samples were left to react for 1 hour at 25°C and then quenched with hydroxylamine (1% final concentration) for 30 min at 37°C. All channels were combined, and the sample was MCW-precipitated. The pellet was washed with methanol, redissolved in 3 M guanidine-HCl, and then diluted to a final concentration of approximately 0.85 M. The sample was digested with trypsin (approximately 35:1 ratio of protein to enzyme) and left to digest overnight at 37°C.

The “neo” N termini generated by trypsin digestion were depleted using HYTANE (*68*) with minor modifications. Briefly, samples were redissolved in Hepes buffer (pH 7.0), then hexadecenal (500 μl, 10 mg/ml) in isopropanol was added along with 20 mM sodium cyanoborohydride, and the reaction left for 5 hours at 50°C. A fresh aliquot of sodium cyanoborohydride was added (20 mM), and then the sample was dried down for 2 hours to a smaller volume, then acidified, and then cleaned with a C_18_ column. The sample was dried (vacuum evaporation) and stored at −20°C before analysis.

All HYTANE samples were injected in duplicate on a Dionex UltiMate 3000 nano-UHPLC coupled to a Q Exactive HF mass spectrometer (Thermo Fisher Scientific, Bremen). The samples were washed on a trap column (Acclaim Pepmap 100 C_18_, 5 mm × 300 μm, 5 μm, 100 Å, Dionex) for 4 min with 3% acetonitrile (ACN)/0.1% trifuoroacetic acid (TFA) at a flow rate of 30 μl/min before peptide separation using an Acclaim PepMap 100 C_18_ analytical column (50 cm × 75 μm, 2 μm, 100 Å, Dionex). A flow rate of 300 nl/min using eluent A [0.05% formic acid (FA)] and eluent B (80% ACN/0.04% FA) was used for gradient separation (180-min gradient, 5 to 40% B). Spray voltage applied on a metal-coated PicoTip emitter (10-μm tip size; New Objective, Woburn, MA, USA) was 1.7 kV, with a source temperature of 250°C. Full-scan MS spectra were acquired between 375 and 1400 mass/charge ratio (*m/z*) at a resolution of 60,000 at *m/z* 200, and the top 10 most intense precursors with charge states greater than 2+ were selected for fragmentation using an isolation window of 1.2 *m/z* and with HCD-normalized collision energies of 33 at a resolution of 30,000. Lock mass (445.120025) and dynamic exclusion (20 and 30 s) were enabled.

The MS raw files were processed by Proteome Discoverer 2.2, and MS/MS spectra were searched using the Sequest HT algorithm against a database containing common contaminants and a mouse database. The enzyme specificity was set to semi-ArgC with two missed cleavages allowed. An MS1 tolerance of 10 parts per million and a MS2 tolerance of 0.02 Da were implemented. Oxidation (15.995 Da) of methionine residues, acetylation (42.011 Da), and TMT-6-plex (229.163 Da) on the peptide N terminus was set as a variable modification, while carbamidomethylation (57.02146 Da) on cysteine residues and TMT on lysine residues was set as a static modification. Technical injection replicates were set as fractions. Normalized, scaled abundance from Proteome Discoverer was exported and log_2_-transformed, and statistical analysis (*t* test) was performed in Perseus (Perseus_1.6.10.43).

### Label-free quantitative proteomics

Label-free quantification was performed on mouse colon samples. One hundred micrograms of each sample was made up to 1% SDS in TEAB buffer. Samples were reduced and alkylated as described above and then quenched with dithiothreitol (DTT). Samples were precipitated onto hydrophilic and hydrophobic Sera-Mag SpeedBeads (carboxylate-modified magnetic beads) using sixfold volume of ethanol. Samples were washed twice with 80% ethanol and then digested with trypsin (1:100, enzyme:protein) overnight at 37°C. Peptides were removed from the beads with the aid of a magnet and then dried and stored at −20°C before analysis. For label-free experiments, only single sample injections were performed, with the same LC settings as for the HYTANE analysis. Samples were measured on a Q Exactive HF mass spectrometer with a mass range from 375 to 1400 *m/z* at a resolution of 60,000 at *m/z* 200. The 10 most intense precursors with charge states greater than 2+ were selected with an isolation window of 1.4 *m/z* and fragmented by HCD with normalized collision energies of 28 at a resolution of 30,000. Lock mass (445.120025) and dynamic exclusion (30 s) were enabled. For label-free experiments, the data were searched with Proteome Discoverer as above except that trypsin specificity was set to full and TMT modifications were excluded in the search. Normalized, scaled abundance from Proteome Discoverer was exported and log_2_-transformed, and statistical analysis (*t* test) was performed in Perseus (Perseus_1.6.10.43). Volcano plots were generated using the GraphPad Prism software. N termini were considered significant if they had a *P* value less than or equal to 0.05 and a log_2_ fold change of ±0.58.

### In-gel digestion

To identify the N termini of recombinant human galectin-3 fragments incubated in the presence and absence of meprin α and meprin β, the corresponding gel bands were excised and then halved to allow for both trypsin and chymotrypsin digestion. Bands were further cut into 2-mm^3^ cubes and destained with 100 mM ammonium bicarbonate (ABC), 30% ACN, 50 mM ABC, and 100% ACN. Samples were reduced with DTT (10 mM) at 65°C for 30 min and then alkylated with iodoacetamide (55 mM final concentration) in the dark at room temperature for 15 min in TEAB buffer. Gels bands were dehydrated with ACN and by vacuum centrifugation and then reductively dimethylated by labeling gel bands with 40 mM formaldehyde in the presence of 20 mM sodium cyanoborohydride (10 mM) in Hepes buffer (200 mM, pH 7) overnight at 25°C. The reaction was quenched by acidification (1% TFA) and then washing with 1% TFA, 50% ACN, and lastly with 100% ACN. Gel bands were dried in the vacuum centrifuge and resuspended in 0.9 M tris-HCl buffer for 2 hours to further quench any residual reagents. Samples were neutralized with acid and washed and dried as described above. Gel bands were digest with either trypsin (40 ng) or chymotrypsin (50 ng) in the presence (human galectin-3) or the absence of heavy water (murine galectin-3; approximately 85% H_2_^18^O) in TEAB buffer (40 mM; 0.5 mM CaCl_2_) overnight at 37°C. Murine galectin-3 samples were only incubated with chymotrypsin. Peptides were extracted from the gel band using 1% FA, 50% ACN, 1% FA, and 100% ACN with the aid of sonication/shaking. Pooled supernatants were dried down by vacuum centrifugation and stored at −20°C until analysis. In-gel digested samples were measured on the Q Exactive with the same LC setup as described above, although with a shorter gradient length. Here, the 10 most intense precursors with charge states greater than 2+ were selected with an isolation window of 2.1 *m*/*z* and fragmented by HCD with normalized collision energies of 28 at a resolution of 17,500. Lock mass (445.120025) and dynamic exclusion (15 s) were enabled. The data were searched with Proteome Discoverer against a database containing common contaminants and the canonical and reviewed human or mouse database, for human and murine galectin-3, respectively. The enzyme specificity was set to semi-ArgC with two missed cleavages allowed (trypsin) or semichymotryptic with up to four missed cleavages allowed. Here, dimethylation (28.031 Da) on lysine residues was set as a static modification, while dimethylation on peptide N termini and heavy oxygen (+2.004 Da) on peptide C termini was set as a variable modification (for example, incubated with heavy water).

### Top-down LC-MS

Gel bands from recombinant human galectin-3 that were (before loading) incubated in the presence and absence of meprin α or meprin α/meprin β were cut into 2-mm^3^ cubes and destained with 100 mM ABC, followed by 50% ACN in 50 mM ABC and then 100% ACN. Gel bands were also dried in the vacuum centrifuge for 5 min. Proteins from gel bands were then passively eluted by shaking in 300 μl of 0.1% SDS and 100 mM ABC in the presence of 2.5 mM TCEP for 30 min. The supernatant was retrieved, another aliquot of SDS/ABC/TCEP buffer was added to the gel bands, and the samples were sonicated for 15 min, followed by shaking for another 15 min. Supernatants were pooled and the samples were lyophilized. Samples were resuspended in 60 μl of water and then cleaned via chloroform/methanol/water precipitation. Samples were left to air-dry and then were frozen at −20°C until analysis.

Top-down samples were run on a Dionex UltiMate 3000 nano-UHPLC coupled to a Fusion Lumos mass spectrometer (Thermo Fisher Scientific, Bremen). The samples were washed on a trap column (C4 column, 5 mm × 300 μm, 5 μm) with 2% ACN/0.05% TFA at a flow rate of 30 μl/min before peptide separation using a C4 analytical column (50 cm × 75 μm). A flow rate of 300 nl/min using eluent A (0.05% FA) and eluent B (80% ACN/0.04% FA) was used for gradient separation (15 to 55% B). Spray voltage applied on a noncoated PicoTip emitter (10-μm tip size; New Objective, Woburn, MA, USA) was 2.3 kV, with a source temperature of 290°C. Full-scan MS spectra were acquired between 500 and 1800 *m/z* at a resolution of 120,000 at *m/z* 400 and 4 microscans. High-feld asymmetric-waveform ion-mobility spectrometr (FAIMS) was used using four compensation voltages (−50, −40, −30, and −20) during analysis (*69*). MS2 were acquired for 1 s with an isolation window of 4 *m/z* and fragmented by collision-induced dissociation with normalized collision energies of 25 at a resolution of 60,000 (also four microscans). Data interpretation was performed manually and using ProSightPD 4.0, with a database containing recombinant human galectin-3.

### STED microscopy

For STED microscopy, 3-μm-thick paraffin-embedded Swiss roll sections of the mouse colon excluding the caecum were preheated at 65°C for 15 min. Afterward, tissue sections were dewaxed in xylene two times, followed by ethanol in decreasing concentrations (100, 95, 70, and 50%). Antigens were retrieved in 10 mM citric buffer (pH 6.0) and boiled for 20 min. After cooling, tissue sections were washed with PBS and incubated with blocking solution [5% fetal bovine serum (FBS)/PBS] for 1 hour at room temperature. Primary antibodies anti-HA (#3724 and #2367, Cell Signaling Technology; 1:1000), anti–meprin α [polyclonal antibody, generated against peptide from the ectodomain (Pineda); 1:1000], anti–galectin-3 (N-terminal, #14-5301-81, eBioscience), and anti–villin-1 (#bsm-54212R, Bioss Antibodies; 1:100) were diluted in blocking solution and incubated with tissue sections in a humid chamber overnight at 4°C. The next day, tissue sections were washed with PBS three times and incubated with a fluorophore-coupled secondary antibody (Thermo Fisher Scientific; 1:300) and 4′,6-diamidino-2-phenylindole (DAPI) (1 μg/ml in PBS; Sigma-Aldrich) diluted in blocking solution for 1 hour in a dark chamber at room temperature. After repeated washing steps, tissue sections were embedded in fluorescent mounting medium (Dako). Sections were analyzed using a multilaser confocal scanning microscope and STED superresolution microscopy. Images were acquired using Facility Line (Abberior Instruments, Göttingen, Germany) with Olympus IX83 microscope (Germany) and Imspector software (Abberior Instruments) in confocal microscopy mode or STED mode.

### Recombinant cleavage assay

For the cleavage of galectin-3 by the meprins, 20 μg of recombinant human galectin-3 (PeproTech) or 10 μg of murine recombinant galectin-3 (R&D Systems) was incubated with or without 5 nM human active recombinant meprin α and/or 5 nM human active recombinant meprin β (*14*) in 20 mM Hepes at 37°C for 2 hours and then stored at −20°C until use. These protein samples were used for SDS-PAGE with subsequent Coomassie staining or for the bacterial agglutination assay.

### Coomassie brilliant blue staining

To visualize proteins in acrylamide gels, a Coomassie brilliant blue staining was carried out. Gels were incubated with Coomassie brilliant blue solution [40% (v/v) methanol, 10% (v/v) acetic acid, and 0.1% (w/v) Coomassie R250 in double-distilled H_2_O] for 1 hour. Then, gels were detained with destaining solution [40% (v/v) methanol and 10% (v/v) acetic acid in double-distilled H_2_O] for several hours until only the protein bands remained blue and, afterward, digitalized or used for in-gel digestion.

### Cell culture and transfection

HEK293T [Deutsche Sammlung von Mikroorganismen und Zellkulturen (DSMZ)] and HEK ADAM10/17^−/−^ cells (ADAM10- and ADAM17-deficient HEK293T, generated by B. Rabe, Institute of Biochemistry, Kiel) were maintained at 37°C under a humidified atmosphere (5% CO_2_) in Dulbecco’s modified Eagles’s medium (DMEM; Thermo Fisher Scientific) supplemented with 10% FBS (Thermo Fisher Scientific), penicillin (100 U/ml), and streptomycin (100 μg/ml) (Thermo Fisher Scientific). For transient transfection, the cells were seeded 24 hours prior at a density of 2.5 × 10^6^ cells per 10-cm cell culture dish. Cells were transfected with 6 μg of plasmid-DNA in total, premixed with polyethyleneimine (1:3) in serum-free medium, and incubated for 30 min at room temperature. Plasmid-DNA with human galectin-3 (pCMV3), human meprin α (pSG5), human meprin β (pSG5), human meprin β–E153A (pSG5), human ADAM17 (codon-optimized, pCMV6), empty vector [pcDNA 3.1 (+)], or plasmids in different combinations was added together with transfection reagent to fresh medium on the cells. After 24 hours, cells were washed with PBS and the medium was changed to serum-free DMEM avoiding meprin β inhibition.

### Fluorogenic peptide-based activity assay

To quantify meprin α and meprin β activity, specific fluorogenic peptide substrates for meprin α or meprin β cleavage were used [(mca)-HVANDPIW-(K-ε-dnp) for meprin α; (mca)-EDEDED-(K-ε-dnp) for meprin β; mca, 7-methyloxycoumarin-4-yl; dnp, 2,4-dinitrophenyl; Genosphere Biotechnologies, Paris, France]. These consisted of a fluorophore (mca), a specific peptide sequence for meprin α (HVANDPIW) or meprin β (EDEDED) and a quencher (dnp). The peptide sequence fits to the cleavage site specificity of meprin α or meprin β (*70*, *71*). For analyzing meprin α and meprin β activity in tissue lysates, 50 to 100 μg of lysate in 100 μl of total volume in a 96-well plate was used. For determination of meprin β activity at the cell surface, HEK293T ADAM10/17^−/−^ cells were transfected 48 hours before. Cells were counted, and 0.3 × 10^6^ cells were plated onto 48-well plates in a total volume of 300 μl. Remaining cells were used for cell lysates and Western blot analysis. Immediately before measurement, 10 μM quenched fluorogenic peptide substrate for meprin α and 50 μM quenched fluorogenic peptide substrate for meprin β were added to cells or tissue lysates. Fluorescence at λ_em_ = 405 nm and λ_ex_ = 320 nm was detected at 37°C every 30 s for 120 min using a spectrophotometer (Tecan, Männedorf, Switzerland). For data analysis, slope of equal linear activity range from three or more individual experiments was used, and all values were normalized to the meprin β single transfection (for the cell surface activity) or to the highest value of the tissue lysate measurement of the proximal colon that was set as 1.

### Ultracentrifugation and trichloroacetic acid precipitation

Cell supernatants were harvested 48 hours after transfection and ultracentrifuged at 186,000*g* for 2 hours at 4°C. To concentrate soluble proteins from cell supernatants, a trichloroacetic acid (TCA) precipitation was used. For this, 1 ml of supernatant was mixed with 10% TCA and incubated for 1 hour at 4°C. After that, supernatant was centrifuged at 13,500*g* for 20 min at 4°C. After discarding the supernatant, 350 μl of precooled acetone (−20°C) was added to the precipitated proteins, and the samples were inverted carefully. After a centrifugation at 13,500*g* for 15 min at 4°C, the acetone was discarded and evaporated completely. Precipitated proteins were then solved in reducing sample buffer, denatured for 10 min at 95°C, and analyzed via SDS-PAGE, NuPAGE, or Western blot.

### Cell surface biotinylation assay

For the cell surface biotinylation assay, transfected cells were washed with ice-cold PBS-CM (0.1 mM CaCl_2_ and 1 mM MgCl_2_ in PBS) and incubated with 3 ml of biotin solution [EZ-Link Sulfo-NHS-SS-Biotin (1 mg/ml; Thermo Fisher Scientific) in PBS-CM] for 30 min at 4°C. Subsequently, the biotin solution was removed, and the cells were incubated with quenching buffer [50 mM tris-HCl (pH 8.0) in PBS-CM] for 10 min. Afterward, cells were washed with ice-cold PBS-CM and lysed in biotinylation lysis buffer [50 mM tris-HCl (pH 7.4), 150 mM NaCl, 1% (v/v) Triton X-100, 0.1% (w/v) SDS, and 1× cOmplete protease inhibitor cocktail (Roche)] for 1 hour. As a lysate control, 300 μg was separated. Five hundred micrograms of lysate was incubated with 50 μl of Pierce Streptavidin Magnetic Beads (Thermo Fisher Scientific), added to the remaining lysate, and incubated overnight at 4°C. After washing the beads with biotinylation lysis buffer, the proteins were removed and denatured by addition of sample buffer [250 mM tris-HCl, 10% (w/v) SDS, 0.5% bromophenol blue, 50% (v/v) glycerol, and 154 mg of DTT (pH 6.8)] for 10 min at 95°C. The samples were analyzed by SDS-PAGE and Western blot analysis.

### Coimmunoprecipitation

Harvested cells were lysed in 700 μl of IP lysis buffer [50 mM tris-HCl (pH 7.4), 120 mM NaCl, 0.5% (v/v) NP-40, and 1× cOmplete protease inhibitor cocktail (Roche)]. As a lysate control and for BCA assay (Thermo Fisher Scientific), 150 μl of the lysate was separated and as lysate control for the SDS-PAGE. The co-IP was done with the Dynabeads Protein G Immunoprecipitation Kit (10007D, Thermo Fisher Scientific) according to the manufacturer’s instructions. For the co-IP, 1 μg of anti–HA-tag antibody (C299F4, Cell Signaling Technology) was used. Protein analysis was performed using SDS-PAGE and Western blot.

### Cell lysis, SDS-PAGE, NuPAGE, and Western blot analysis

For SDS-PAGE and Western blot analysis, cells were harvested 48 hours after transfection, washed with PBS, and then incubated for 1 hour at 4°C with lysis buffer [1× cOmplete protease inhibitor cocktail (Roche) and 1% (v/v) Triton X-100 in PBS (pH 7.4)]. The lysate was centrifuged at 13,500*g* at 4°C, and the cell debris was discarded. The protein concentration in the lysate was determined using the Pierce BCA Protein Assay Kit (Thermo Fisher Scientific) according to the manufacturer’s instructions. As preparation for the SDS-PAGE or NuPAGE, the samples were incubated with sample buffer [250 mM tris-HCl, 10% (w/v) SDS, 0.5% bromophenol blue (Merck), 50% (v/v) glycerol, and 154 mg of DTT (pH 6.8)] for 10 min at 95°C. The protein separation was performed by SDS-PAGE (129 V) using the Laemmli conditions in the Mini-PROTEAN system (Bio-Rad) or the NuPAGE system (90 V) (Thermo Fisher Scientific). The Western blot analysis was accomplished with a Tank-Blot system (Bio-Rad) onto polyvinylidene fluoride (Millipore) membranes (0.8 A for 2 hours at 4°C). Afterward, the membranes were blocked with 5% milk (w/v) in tris-buffered saline (TBS) or tris-buffered saline with tween-20 (TBS-T) for 1 hour at room temperature. The primary antibodies against meprin α and meprin β [polyclonal antibodies, generated against peptide from the ectodomain (Pineda)], HA-tag (C299F4, #3724, Cell Signaling Technology), galectin-3 (M3/38, #126702, BioLegend), galectin-3 (N-terminal, #14-5301-81, eBioscience), ADAM17 (A300D) (*72*), transferrin receptor (ab84036, Abcam), and glyceraldehyde-3-phosphate dehydrogenase (GAPDH) (14C10, #2118, Cell Signaling Technology) were incubated using a dilution of 1:1000 in 5% milk (w/v) in TBS with the membrane overnight at 4°C. Horseradish peroxidase–conjugated secondary antibodies (1:10.000; Jackson ImmunoResearch) were diluted in 10% milk (w/v) in TBS and incubated with the membranes for 1 hour at room temperature. The chemiluminescence signal was detected in the Intelligent Dark Box (LAS-3000, Fujifilm) using the SuperSignal West Pico PLUS chemiluminescent substrate or SuperSignal West Femto Substrate with maximum sensitivity (Thermo Fisher Scientific) according to the manufacturer’s instructions.

### Native PAGE

For the native PAGE, the samples were gently mixed with nonreducing sample buffer [62.5 mM tris-HCl, 1.0% bromophenol blue (Merck), and 25% (v/v) glycerol (pH 7.0)]. Because of the isoelectric point of galectin-3 (pI, ~8.58) (*73*), the protein separation was performed using the Mini-PROTEAN system {120 V, running buffer [25 mM tris-HCl and 192 mM glycine (pH 7.0)], Bio-Rad} without using SDS and a pH of 7.0 from the anode to the cathode. To visualize the protein, the gel was afterward Coomassie brilliant blue–stained.

### Cell differentiation and treatment

For differentiation, Caco-2 (DSMZ) cells were seeded on polyethylene terephthalate filters (TC-Inserts, 12-well, 0.4 μm, translucent, Sarstedt) at a density of 3.85 × 10^5^ cells per insert in DMEM (Thermo Fisher Scientific) supplemented with 20% FBS (Thermo Fisher Scientific), penicillin (100 U/ml) and streptomycin (100 μg/ml) (Thermo Fisher Scientific) in both apical and basolateral compartments for 3 days to allow the formation of a confluent cell monolayer. From day 3 after seeding, cells were transferred to complete medium in both compartments, supplemented with 20% FBS only in the basolateral compartment and allowed to differentiate for 21 days with regular medium changes two to three times a week. For treatment, Caco-2 cells were washed with PBS on day 17, and medium was changed to serum-free DMEM without phenol red (Sigma-Aldrich) in both compartments. For the meprin inhibitor actinonin (Merck), 3 μM was added daily to the basolateral and the apical compartment until day 21. For the bacterial meprin activator RgpB (*7*) (purified protease from *P. gingivalis* kindly provided by J. Potempa, University of Louisville), 5 nM was added to both compartments on day 20. Cells were harvested on day 21.

### Microscopy of differentiated Caco-2 cells

Morphological evaluation of Caco-2 cell differentiation was performed by F-actin, E-cadherin, and villin localization as well as nuclear staining. On day 21, Caco-2 were washed with 0.2% (w/v) saponin in PBS and fixed in 4% (w/v) paraformaldehyde in PBS for 20 min at room temperature. Subsequently, cells were washed with 0.2% (w/v) saponin in PBS and were permeabilized with 0.12% (w/v) glycine and 0.2% (w/v) saponin in PBS for 10 min. Afterward, cells were washed with 0.2% (w/v) saponin in PBS and incubated with blocking solution [10% FBS and 0.2% (w/v) saponin in PBS] for 30 min at room temperature. Primary antibodies anti-ZO-1 (D6L1E, #13663, Cell Signaling Technology; 1:400), anti-villin (PA5-17290, Thermo Fisher Scientific; 1:100), and anti-E-cadherin (610405, BD Transduction Laboratories; 1:500) were diluted in blocking solution and incubated in a humid chamber for 1 hour. Afterward, the cells were washed three times with 0.2% (w/v) saponin in PBS and incubated with a fluorophore-coupled secondary antibody (Thermo Fisher Scientific; 1:300) and Phalloidin–iFluor 488 Reagent (ab176753, Abcam; 1:1000) diluted in blocking solution for 1 hour in a dark chamber at room temperature. After repeated washing steps, cells were stained and mounted using with 15 μl of Mowiol/DABCO solution [17% (w/v) Mowiol, 33% (v/v) glycerol, 1,4 – Diazobicyclo[2.2.2.]octan (DABCO) (50 mg/ml), and DAPI (1 μg/ml)] preheated to 60°C. Immunofluorescence analysis was performed using a confocal laser-scanning microscope FV1000 (Olympus, Hamburg). Serial optical sections were processed with FluoView 04.02 (Olympus, Hamburg).

### CRISPR-Cas9 genome editing

For CRISPR-Cas9 genome editing, multiguide RNA (mgRNA) synthesized by Synthego targeting human *MEP1A* or *MEP1B* and recombinant *Streptococcus pyogenes* Cas9 nuclease 2NLS (Synthego) were used to generate isogenic *MEP1A* and *MEP1B* KO cells. Caco-2 cells were transfected with 300 pmol of mgRNA and 60 pmol of Cas9 by Amaxa nucleofection (program: CM-130, Lonza, Cologne, Germany) using the SF Cell Line 4D-Nucleofector X Kit S (Lonza Cologne AG, Cologne, Germany) according to the manufacturer’s instructions. Immediately after electroporation, single-cell seeding in 96-well plates was performed to obtain single-cell clones. Genotyping was performed on extracted genomic DNA (GeneJET Genomic DNA Purification Kit, Thermo Fisher Scientific) using DreamTaq PCR reagents (Thermo Fisher Scientific) and the following oligonucleotides: *MEP1A^−/−^*, 5′-CCCCATCTTTGGACCATTAGC-3′ (forward) and 5′-CCATTCCTTGAGGCCCTTATCT-3′ (reverse); *MEP1B^−/−^*, 5′-GACTAGGCAGTGGCGATTCTG-3′ (forward) and 5′-CCACAGACTCCGTTCCACATA-3′ (reverse).

### mRNA isolation, cDNA transcription, and qRT-PCR

RNA was isolated using NucleoSpin RNA Isolation Kit (Macherey-Nagel) according to the manufacturer’s instructions. Afterward, 1 μg of total mRNA was transcribed into stable cDNA by using RevertAid First Strand cDNA Synthesis Kit (Thermo Fisher Scientific). For qRT-PCR analysis, cDNA was amplified in the LightCycler480 II Real-Time PCR System (Roche Applied Science) according to the manufacturer’s instructions. All specific primers are listed in Table 1 below. Relative amounts of the target gene were normalized to the relative mRNA levels of gapdh.

**Table 1.** Specific primers for quantification of *LGALS3*, *MEP1A*, and *MEP1B* via qPCR.

Forward primer	Reverse primer	Forward primer sequence (5′-3′)	Reverse primer sequence (5′-3′)	*T*_m_ (°C)	Target
Human *LGALS3* #3 For	Human *LGALS3* #3 Rev	CTTCTGGACAGCCAAGTGC	AAAGGCAGGTTATAAGGCACAA	60.0	*LGALS3*
Human *MEP1A* #34 For	Human *MEP1A* #34 Rev	CTTGTTGGGACAATGCACAG	GGGTAAAGAATCCGAGACTCC	60.0	*MEP1A*
Human *MEP1B* #68 For	Human *MEP1B* #68 Rev	CAGTGACTCTGATCTCCTAAAGTTGA	TGCACGAGTCCATAAAACTCA	60.0	*MEP1B*
Human *GAPDH* #60 For	Human *GAPDH* #60 Rev	AGCCACATCGCTCAGACAC	GCCCAATACGACCAAATCC	60.0	*GAPDH*

### Bacterial agglutination assay

For the bacterial agglutination assay the bacterial strains (*E. faecalis* #29212, ATCC; *E. coli* #25922, ATCC; *K. pneumoniae* #13882 and #13883, ATCC; *P. aeruginosa* #27853, ATCC) were plated the day before use from frozen glycerol stocks and cultured on blood agar at 37°C for 24 hours [Columbia-Agar with 7% sheep blood (Thermo Fisher Scientific)]. Bacteria were then harvested by scraping and rinsing off the plate and suspending the colony material in 20 mM Hepes (pH 7.2). The cell suspension was washed by centrifugation at 4500*g* for 5 min at room temperature. The supernatant was discarded, and the cells resuspended in 20 mM Hepes. This washing step was performed twice to remove residues from the blood agar. Bacteria suspension was then diluted in 20 mM Hepes until the optical density at 600 nm (OD_600_) was in a range of 0.8 to 1.0. The diluted bacteria suspension was then used directly for the agglutination assay. Fifty microliters of the respective bacteria suspension was transferred into the center of wells of a 96-well round-bottom plate. After addition of the preincubated recombinant protein samples (as described in the “Recombinant cleavage assay” section), the content of the wells was gently mixed by pipetting up and down. Once the 96-well plates were incubated at room temperature for 24 hours, the presence or absence of bacterial agglutination was examined by visual observation and documented by photography. The area of nonagglutinated bacteria was calculated using ImageJ Fiji. All values were normalized to the control (Hepes buffer), while the control corresponds to 0 and maximum agglutination to 1.

### Fecal microbiome analysis

For fecal microbiome analysis, female wild-type, *mep1a/b^−/−^*, and *lgals3^−/−^* mice (*n* = 6 of each strain) were cohoused for more than 4 weeks. Afterward, feces collection was accomplished using the TransnetYX microbiome sample collection kits containing barcoded sample collection tubes (TransnetYX Microbiome, TransnetYX, Cordova, TN, USA). Two fecal pellets from each mouse were placed in separate tubes containing DNA stabilization buffer to ensure reproducibility, stability, and traceability and shipped for DNA extraction, library preparation, and sequencing (TransnetYX). The samples were analyzed by TransnetYX using shallow shotgun whole-genome sequencing. Raw data (in the form of FASTQ files) were analyzed using the One Codex analysis software and analyzed against the One Codex database consisting of >115,000 whole microbial reference genomes. The relative abundance of each microbial species is estimated on the basis of the depth and coverage of sequencing across every available reference genome.

### Ex vivo mucus characterization

#### Mucus penetrability and mucus structure investigation

Distal colon mucus properties were examined in wild-type and *lgals3^−/−^* as described previously (*47*, *74*). Briefly, the mice were anesthetized with isoflurane, euthanized by cervical dislocation and the abdomen opened. The distal part of the large intestine was dissected and flushed with ice-cold Krebs transport solution. The tissue was opened longitudinally, and the longitudinal muscle layer removed before mounting the epithelium in a chilled horizontal Ussing chamber with the basolateral chamber filled with ice-cold Krebs transport solution. The tissue and mucus were stained by apical application of SYTO 9 Green Fluorescent Nucleic Acid Stain (25 μM; Thermo Fisher Scientific) and Dylight 649–conjugated *Ulex europaeus* agglutinin I (UEA-I) (50 μg/ml; Vector Laboratories) and rhodamine-labeled wheat germ agglutinin (WGA) (50 μg/ml; Thermo Fisher Scientific) in a total volume of 50 μl of ice-cold Krebs transport solution for 15 min on ice. After removal of the apical staining solution and gentle rinsing, 1-μm FluoSpheres carboxylate-modified microspheres (365/415) (Thermo Fisher Scientific) diluted 1:10 in Krebs transport solution were applied and left to sediment 5 min before gentle rinsing and imaging using an LSM700 Axio Examiner Z.1 confocal microscope with Plan-Apochromat ×20/1.0 dissolved inorganic carbon water objective and the ZEN 2010 software. Confocal image analysis and processing were performed in Imaris (version 9.5.0, Bitplane AG). Quantification of bead penetration and mucus thickness was performed as previously described (*75*).

#### Mucus growth analysis

For mucus growth analysis, the tissue was mounted in a horizontal perfusion chamber with basolateral perfusion of Krebs solution supplemented with glucose and mannitol supplemented static Krebs solution on the apical side at 37°C. The mucus was visualized by application of charcoal particles, and the mucus thickness was measured using a glass needle connected to a micromanipulator at *t* = 0, 15, and 30 min after mounting. The growth rate was calculated as the delta mucus thickness divided by time.

### Bacterial 16*S* rRNA gene quantification by qPCR

Stool samples for bacterial 16*S* quantification was collected from wild-type, *lgals3^−/−^*, and *mep1a/b^−/−^* mice that had been cohoused for more than 6 weeks. The samples were collected under aseptic conditions from approximately 2 cm of the distal colon. The colon was flushed and rinsed with 5 ml of ice-cold 0.22-μm filter sterilized PBS, and stool samples were collected from the flushed material.

DNA was extracted using QIAamp PowerFecal Pro DNA kit (QIAGEN) according to the manufacturer’s instructions. Sample homogenization was achieved by four rounds of bead-beating performed on a Fasp-Prep System (MPBio) at 4.5 m/s for 40 s with cooling on ice between beating. DNA concentration was determined using Nanodrop.

Quantification of bacterial 16*S* by qPCR was performed using a maximum of 50 ng of DNA template with SsoFast EvaGreen Supermix (Bio-Rad) and 0.3 μM specific primers as listed in Table 2. Reactions were run on a CFX96 platform (Bio-Rad) and analyzed using CFX Manager (version 3.1). For relative quantification of specific bacterial taxa (Firmicutes, Bacteroidetes, and Proteobacteria), *C*_q_ values from qPCR using taxon-specific primer pairs were normalized to values obtained using universal primers using the ΔΔ*C*_q_ method.

**Table 2.** Specific primers for quantification of bacterial 16*S* by qPCR.

Forward primer	Reverse primer	Forward primer sequence (5′-3′)	Reverse primer sequence (5′-3′)	*T*_m_	Target
926F	1062R	AAACTCAAAKGAATTGACGG	CTCACRRCACGAGCTGAC	61.5	Universal
928F-Firm	1040FirmR	TGAAACTYAAAGGAATTGACG	ACCATGCACCACCTGTC	61.5	Firmicutes
798cfbF	cfb967R	CRAACAGGATTAGATACCCT	GGTAAGGTTCCTCGCGTAT	61.5	Bacteroidetes
1080gF	g1202R	TCGTCAGCTCGTGTYGTGA	CGTAAGGGCCATGATG	61.5	Proteobacteria

### *C. rodentium* infection

Animal experiments for the *C. rodentium* infection were carried out with healthy age-matched 8- to 10-week-old female C57BL/6 J mice in accordance with current guidelines of the European Union after approval by the Institutional Review Board of the Medical University of Vienna and the Austrian Ministry of Sciences (protocol ID GZ-2022-0.386.466). Mice were maintained in specific pathogen-free environment at the Core Facility Laboratory Animal Breeding and Husbandry of the Medical University of Vienna. Animals from different litters were randomly intermixed and cohoused for at least 2 weeks before experiments to reduce potential cage-specific, microbiome-related effects. *C. rodentium* (strain DBS100) was provided by G. Boeckxstaens, KU Leuven, Belgium. For preparation of bacterial inoculate, frozen stocks were streaked on MacConkey agar plates (Sigma-Aldrich) and incubated overnight at 37°C. Ten milliliters of Luria-Bertani broth (LB) medium were inoculated from a single bacterial colony and incubated overnight at 37°C with shaking at 180 rpm. The overnight liquid culture was used to inoculate fresh LB medium in an Erlenmeyer flask at a dilution of 1:200. Bacteria were harvested after culture at 37°C at 180 rpm at an OD_600_ of 0.5 to 0.9 by centrifugation for 5 min at 1800*g* at 4°C, washed once with endotoxin-free PBS (Gibco), and resuspended in endotoxin-free PBS at 5 × 10^9^ colony-forming units (CFUs)/ml. Before infection, serial dilutions of the inoculum were plated on MacConkey agar to quantify the titer (viable bacteria). For mouse infection, animals were transferred to biosafety level 2 environment and allowed to acclimatize for at least 5 days. Mice were infected with 10^9^ CFUs of *C. rodentium* (or mock-infected with endotoxin-free PBS) by oral gavage using a dosing cannula (Harvard Apparatus) (*76*).

On day 7 after infection, animals were euthanized, and the colon was collected and kept on ice. Colon tissue was homogenized in RPMI 1640 medium (Gibco) using a Precellys homogenizer (Bertin) and metal beads. Tissue lysate was mixed 1:1 with lysate buffer [300 mM NaCl, 30 mM Trizma base, 2 mM MgCl_2_, and 2 mM CaCl_2_ (pH 7.4); all chemicals from Sigma-Aldrich] containing protease inhibitor cocktail (P8340, Sigma-Aldrich) and incubated 30 min at 4°C. Following 15 min of centrifugation at 1500*g* and 4°C, supernatant was collected and stored at −80°C.

### Human biopsies

Patients with UC and healthy controls gave written informed consent before colonoscopy for biopsy collection. Approval was granted by the ethics committee of the medical faculty of Kiel University (B231/98). In total, four severely inflamed colonic intestinal biopsies from patients with CU and from four healthy controls were collected and shock-frozen in liquid nitrogen. Frozen biopsies were ground in liquid nitrogen to a fine powder, resuspended in 150 μl of EDTA-free lysis buffer [1× cOmplete protease inhibitor cocktail, EDTA-free (Roche), 1% (v/v) Triton X-100, and PBS (pH 7.4)] and incubated for 45 min at 4°C. The lysate was centrifuged for 15 min at 15,000*g* at 4°C, and protein amount was determined using the Pierce BCA Protein Assay Kit (Thermo Fisher Scientific) following the manufacturer’s instructions.

### Quantification and statistical analysis

All statistical analyzes were performed with the GraphPad Prism 7.04 software. In case of two datasets, an unpaired *t* test was applied. The examination of datasets of more datasets was accomplished with a one- or two-way analysis of variance (ANOVA), followed by a Tukey posttest (*P < 0.05; **P < 0.01; ***P < 0.001). Values are expressed as means ± SD. The figures were created with BioRender.com.

### Data and software availability

The MS-based proteomics data have been uploaded to the ProteomeXchange Consortium via the PRIDE (*77*) partner repository with the dataset identifier PXD037000.

## Notes

View/request a protocol for this paper from *Bio-protocol*.

## References

[1] M. E. Johansson, H. Sjövall, G. C. Hansson, The gastrointestinal mucus system in health and disease. Nat. Rev. Gastroenterol. Hepatol. 10, 352–361 (2013). 23478383 10.1038/nrgastro.2013.35 PMC3758667

[2] A. J. Macpherson, E. Slack, M. B. Geuking, K. D. McCoy, The mucosal firewalls against commensal intestinal microbes. Semin. Immunopathol. 31, 145–149 (2009). 19707762 10.1007/s00281-009-0174-3

[3] M. E. Johansson, J. K. Gustafsson, J. Holmén-Larsson, K. S. Jabbar, L. Xia, H. Xu, F. K. Ghishan, F. A. Carvalho, A. T. Gewirtz, H. Sjövall, G. C. Hansson, Bacteria penetrate the normally impenetrable inner colon mucus layer in both murine colitis models and patients with ulcerative colitis. Gut 63, 281–291 (2014). 23426893 10.1136/gutjnl-2012-303207 PMC3740207

[4] A. L. Haber, M. Biton, N. Rogel, R. H. Herbst, K. Shekhar, C. Smillie, G. Burgin, T. M. Delorey, M. R. Howitt, Y. Katz, A single-cell survey of the small intestinal epithelium. Nature 551, 333–339 (2017). 29144463 10.1038/nature24489 PMC6022292

[5] S. Banerjee, G. Jin, S. G. Bradley, G. L. Matters, R. D. Gailey, J. M. Crisman, J. S. Bond, Balance of meprin A and B in mice affects the progression of experimental inflammatory bowel disease. Am. J. Physiol. Gastrointest. Liver Physiol. 300, G273–G282 (2011). 21071511 10.1152/ajpgi.00504.2009 PMC3043644

[6] S. Banerjee, B. Oneda, L. Yap, D. Jewell, G. Matters, L. Fitzpatrick, F. Seibold, E.-E. Sterchi, T. Ahmad, D. Lottaz, J. S. Bond, MEP1A allele for meprin a metalloprotease is a susceptibility gene for inflammatory bowel disease. Mucosal Immunol. 2, 220–231 (2009). 19262505 10.1038/mi.2009.3 PMC2670347

[7] F. Peters, F. Scharfenberg, C. Colmorgen, F. Armbrust, R. Wichert, P. Arnold, B. Potempa, J. Potempa, C. U. Pietrzik, R. Häsler, P. Rosenstiel, C. Becker-Pauly, Tethering soluble meprin α in an enzyme complex to the cell surface affects IBD-associated genes. FASEB J. 33, 7490–7504 (2019). 30916990 10.1096/fj.201802391R PMC6529335

[8] A. Schütte, A. Ermund, C. Becker-Pauly, M. E. Johansson, A. M. Rodriguez-Pineiro, F. Bäckhed, S. Müller, D. Lottaz, J. S. Bond, G. C. Hansson, Microbial-induced meprin β cleavage in MUC2 mucin and a functional CFTR channel are required to release anchored small intestinal mucus. Proc. Natl. Acad. Sci. U.S.A. 111, 12396–12401 (2014). 25114233 10.1073/pnas.1407597111 PMC4151749

[9] R. Wichert, A. Ermund, S. Schmidt, M. Schweinlin, M. Ksiazek, P. Arnold, K. Knittler, F. Wilkens, B. Potempa, B. Rabe, M. Stirnberg, R. Lucius, J. W. Bartsch, S. Nikolaus, M. Falk-Paulsen, P. Rosenstiel, M. Metzger, S. Rose-John, J. Potempa, G. C. Hansson, P. J. Dempsey, C. Becker-Pauly, Mucus detachment by host metalloprotease meprin β requires shedding of its inactive pro-form, which is abrogated by the pathogenic protease RgpB. Cell Rep. 21, 2090–2103 (2017). 29166602 10.1016/j.celrep.2017.10.087

[10] K. Barnes, J. Ingram, A. J. Kenny, Proteins of the kidney microvillar membrane. Structural and immunochemical properties of rat endopeptidase-2 and its immunohistochemical localization in tissues of rat and mouse. Biochem. J. 264, 335–346 (1989). 2690825 10.1042/bj2640335 PMC1133587

[11] R. J. Beynon, J. Shannon, J. S. Bond, Purification and characterization of a metallo-endoproteinase from mouse kidney. Biochem. J. 199, 591–598 (1981). 7041888 10.1042/bj1990591 PMC1163414

[12] E. E. Sterchi, J. R. Green, M. J. Lentze, Non-pancreatic hydrolysis of *N*-benzoyl-l-tyrosyl-*p*-aminobenzoic acid (PABA-peptide) in the human small intestine. Clin. Sci. 62, 557–560 (1982). 10.1042/cs0620557 7042181

[13] C. Bayly-Jones, C. J. Lupton, C. Fritz, H. Venugopal, D. Ramsbeck, M. Wermann, C. Jager, A. de Marco, S. Schilling, D. Schlenzig, J. C. Whisstock, Helical ultrastructure of the oncogenic metalloprotease meprin α in complex with a small molecule hydroxamate inhibitor. Nat. Commun. 13, 6178 (2022). 36261433 10.1038/s41467-022-33893-7 PMC9581967

[14] C. Becker, M.-N. Kruse, K. A. Slotty, D. Köhler, J. R. Harris, S. Rösmann, E. E. Sterchi, W. Stöcker, Differences in the activation mechanism between the α and β subunits of human meprin. Biol. Chem. 384, 825–831 (2003). 12817480 10.1515/BC.2003.092

[15] G. P. Bertenshaw, M. T. Norcum, J. S. Bond, Structure of homo- and hetero-oligomeric meprin metalloproteases. Dimers, tetramers, and high molecular mass multimers. J. Biol. Chem. 278, 2522–2532 (2003). 12399461 10.1074/jbc.M208808200

[16] D. Hahn, A. Pischitzis, S. Roesmann, M. K. Hansen, B. Leuenberger, U. Luginbuehl, E. E. Sterchi, Phorbol 12-myristate 13-acetate-induced ectodomain shedding and phosphorylation of the human meprinβ metalloprotease. J. Biol. Chem. 278, 42829–42839 (2003). 12941954 10.1074/jbc.M211169200

[17] C. Herzog, R. S. Haun, A. Ludwig, S. V. Shah, G. P. Kaushal, ADAM10 is the major sheddase responsible for the release of membrane-associated meprin a. J. Biol. Chem. 289, 13308–13322 (2014). 24662289 10.1074/jbc.M114.559088 PMC4036340

[18] T. Jefferson, C. Bellac, V. V. Metz, C. Broder, J. Hedrich, A. Ohler, W. Maier, V. Magdolen, E. Sterchi, J. S. Bond, A. Jayakumar, H. Traupe, A. Chalaris, S. Rose-John, C. U. Pietrzik, R. Postina, C. M. Overall, C. Becker-Pauly, The substrate degradome of meprin metalloproteases reveals an unexpected proteolytic link between meprin β and ADAM10. Cell. Mol. Life Sci. 70, 309–333 (2013). 22940918 10.1007/s00018-012-1106-2 PMC3535375

[19] L. Werny, A. Grogro, K. Bickenbach, C. Bülck, F. Armbrust, T. Koudelka, K. Pathak, F. Scharfenberg, M. Sammel, F. Sheikhouny, A. Tholey, S. Linder, C. Becker-Pauly, MT1-MMP and ADAM10/17 exhibit a remarkable overlap of shedding properties. FEBS J. 290, 93–111 (2023). 35944080 10.1111/febs.16586

[20] J. Hirabayashi, K.-i. Kasai, The family of metazoan metal-independent β-galactoside-binding lectins: Structure, function and molecular evolution. Glycobiology 3, 297–304 (1993). 8400545 10.1093/glycob/3.4.297

[21] O. Yuko, L. Hakon, S. Yasuhiko, K. Ken-ichi, S. H. Barondes, Human breast carcinoma cDNA encoding a galactoside-binding lectin homologous to mouse mac-2 antigen. Gene 99, 279–283 (1991). 2022338 10.1016/0378-1119(91)90139-3

[22] J. Nio-Kobayashi, H. Takahashi-Iwanaga, T. Iwanaga, Immunohistochemical localization of six galectin subtypes in the mouse digestive tract. J. Histochem. Cytochem. 57, 41–50 (2009). 18796404 10.1369/jhc.2008.952317 PMC2605710

[23] R. S. Bresalier, J. C. Byrd, L. Wang, A. Raz, Colon cancer mucin: A new ligand for the beta-galactoside-binding protein galectin-3. Cancer Res. 56, 4354–4357 (1996). 8813123

[24] S. K. Gupta, S. Masinick, M. Garrett, L. D. Hazlett, *Pseudomonas aeruginosa* lipopolysaccharide binds galectin-3 and other human corneal epithelial proteins. Infect. Immun. 65, 2747–2753 (1997). 9199445 10.1128/iai.65.7.2747-2753.1997 PMC175387

[25] D. Kavanaugh, M. Kane, L. Joshi, R. M. Hickey, Detection of galectin-3 interaction with commensal bacteria. Appl. Environ. Microbiol. 79, 3507–3510 (2013). 23524672 10.1128/AEM.03694-12 PMC3648028

[26] A. Mey, H. Leffler, Z. Hmama, G. Normier, J.-P. Revillard, The animal lectin galectin-3 interacts with bacterial lipopolysaccharides via two independent sites. J. Immunol. 156, 1572–1577 (1996). 8568262

[27] A.-M. Park, S. Hagiwara, D. K. Hsu, F.-T. Liu, O. Yoshie, Galectin-3 plays an important role in innate immunity to gastric infection by *Helicobacter pylori*. Infect. Immun. 84, 1184–1193 (2016). 26857579 10.1128/IAI.01299-15 PMC4807496

[28] L. Frol’ová, K. Smetana, D. Borovská, A. Kitanovičová, K. Klimešová, I. Janatková, K. Malíčková, M. Lukáš, P. Drastich, Z. Beneš, Detection of galectin-3 in patients with inflammatory bowel diseases: New serum marker of active forms of IBD? Inflamm. Res. 58, 503–512 (2009). 19271150 10.1007/s00011-009-0016-8

[29] E. Jensen-Jarolim, R. Gscheidlinger, G. Oberhuber, C. Neuchrist, T. Lucas, G. Bises, C. Radauer, M. Willheim, O. Scheiner, F.-T. Liu, G. Boltz-Nitulescu, The constitutive expression of galectin-3 is downregulated in the intestinal epithelia of Crohn’s disease patients, and tumour necrosis factor alpha decreases the level of galectin-3-specific mRNA in HCT-8 cells. Eur. J. Gastroenterol. Hepatol. 14, 145–152 (2002). 11981338 10.1097/00042737-200202000-00008

[30] E. Lippert, M. Stieber-Gunckel, N. Dunger, W. Falk, F. Obermeier, C. Kunst, Galectin-3 modulates experimental colitis. Digestion 92, 45–53 (2015). 26202676 10.1159/000431312

[31] H.-F. Tsai, C.-S. Wu, Y.-L. Chen, H.-J. Liao, I.-T. Chyuan, P.-N. Hsu, Galectin-3 suppresses mucosal inflammation and reduces disease severity in experimental colitis. J. Mol. Med. 94, 545–556 (2016). 26631140 10.1007/s00109-015-1368-x

[32] Y. Zhou, B. Zhou, L. Pache, M. Chang, A. H. Khodabakhshi, O. Tanaseichuk, C. Benner, S. K. Chanda, Metascape provides a biologist-oriented resource for the analysis of systems-level datasets. Nat. Commun. 10, 1523 (2019). 30944313 10.1038/s41467-019-09234-6 PMC6447622

[33] M. Zhang, L. Liu, X. Lin, Y. Wang, Y. Li, Q. Guo, S. Li, Y. Sun, X. Tao, D. Zhang, X. Lv, L. Zheng, L. Ge, A translocation pathway for vesicle-mediated unconventional protein secretion. Cell 181, 637–652.e15 (2020). 32272059 10.1016/j.cell.2020.03.031

[34] J. Fogh, J. M. Fogh, T. Orfeo, One hundred and twenty-seven cultured human tumor cell lines producing tumors in nude mice. J. Natl. Cancer Inst. 59, 221–226 (1977). 327080 10.1093/jnci/59.1.221

[35] M. Pinto, Enterocyte-like differentiation and polarization of the human colon carcinoma cell line Caco-2 in culture. Biol. Cell 47, 323–330 (1983).

[36] M.-N. Kruse, C. Becker, D. Lottaz, D. Köhler, I. Yiallouros, H.-W. Krell, E. E. Sterchi, W. Stöcker, Human meprin alpha and beta homo-oligomers: Cleavage of basement membrane proteins and sensitivity to metalloprotease inhibitors. Biochem. J. 378, 383–389 (2004). 14594449 10.1042/BJ20031163 PMC1223953

[37] D. K. Hsu, R. I. Zuberi, F.-T. Liu, Biochemical and biophysical characterization of human recombinant IgE-binding protein, an S-type animal lectin. J. Biol. Chem. 267, 14167–14174 (1992). 1629216

[38] S. M. Massa, D. N. Cooper, H. Leffler, S. H. Barondes, L-29, an endogenous lectin, binds to glycoconjugate ligands with positive cooperativity. Biochemistry 32, 260–267 (1993). 8418845 10.1021/bi00052a033

[39] B. Mehul, S. Bawumia, S. R. Martin, R. C. Hughes, Structure of baby hamster kidney carbohydrate-binding protein CBP30, an S-type animal lectin. J. Biol. Chem. 269, 18250–18258 (1994). 8027086

[40] J. Ochieng, D. Platt, L. Tait, V. Hogan, T. Raz, P. Carmi, A. Raz, Structure-function relationship of a recombinant human galactoside-binding protein. Biochemistry 32, 4455–4460 (1993). 8476870 10.1021/bi00067a038

[41] N. Agrwal, Q. Sun, S.-Y. Wang, J. Wang, Carbohydrate-binding protein 35. I. Properties of the recombinant polypeptide and the individuality of the domains. J. Biol. Chem. 268, 14932–14939 (1993). 8325870

[42] J. Herrmann, C. Turck, R. E. Atchison, M. Huflejt, L. Poulter, M. Gitt, A. Burlingame, S. Barondes, H. Leffler, Primary structure of the soluble lactose binding lectin L-29 from rat and dog and interaction of its non-collagenous proline-, glycine-, tyrosine-rich sequence with bacterial and tissue collagenase. J. Biol. Chem. 268, 26704–26711 (1993). 8253805

[43] J. Ochieng, R. Fridman, P. Nangia-Makker, D. E. Kleiner, L. A. Liotta, W. G. Stetler-Stevenson, A. Raz, Galectin-3 is a novel substrate for human matrix metalloproteinases-2 and -9. Biochemistry 33, 14109–14114 (1994). 7947821 10.1021/bi00251a020

[44] J. Ochieng, B. Green, S. Evans, O. James, P. Warfield, Modulation of the biological functions of galectin-3 by matrix metalloproteinases. Biochim. Biophys. Acta 1379, 97–106 (1998). 9468337 10.1016/s0304-4165(97)00086-x

[45] A. Raz, G. Pazerini, P. Carmi, Identification of the metastasis-associated, galactoside-binding lectin as a chimeric gene product with homology to an IgE-binding protein. Cancer Res. 49, 3489–3493 (1989). 2525069

[46] S. Song, J. C. Byrd, N. Mazurek, K. Liu, J. S. Koo, R. S. Bresalier, Galectin-3 modulates MUC2 mucin expression in human colon cancer cells at the level of transcription via AP-1 activation. Gastroenterology 129, 1581–1591 (2005). 16285957 10.1053/j.gastro.2005.09.002

[47] J. K. Gustafsson, A. Ermund, M. E. Johansson, A. Schütte, G. C. Hansson, H. Sjövall, An ex vivo method for studying mucus formation, properties, and thickness in human colonic biopsies and mouse small and large intestinal explants. Am. J. Physiol. Gastrointest. Liver Physiol. 302, G430–G438 (2012). 22159279 10.1152/ajpgi.00405.2011 PMC4073982

[48] A. I. Markowska, K. C. Jefferies, N. Panjwani, Galectin-3 protein modulates cell surface expression and activation of vascular endothelial growth factor receptor 2 in human endothelial cells. J. Biol. Chem. 286, 29913–29921 (2011). 21715322 10.1074/jbc.M111.226423 PMC3191032

[49] S. P. Rosshart, J. Herz, B. G. Vassallo, A. Hunter, M. K. Wall, J. H. Badger, J. A. McCulloch, D. G. Anastasakis, A. A. Sarshad, I. Leonardi, N. Collins, J. A. Blatter, S.-J. Han, S. Tamoutounour, S. Potapova, M. B. F. St. Claire, W. Yuan, S. K. Sen, M. S. Dreier, B. Hild, M. Hafner, D. Wang, I. D. Iliev, Y. Belkaid, G. Trinchieri, B. Rehermann, Laboratory mice born to wild mice have natural microbiota and model human immune responses. Science 365, eaaw4361 (2019). 31371577 10.1126/science.aaw4361 PMC7377314

[50] A. D. Kostic, R. J. Xavier, D. Gevers, The microbiome in inflammatory bowel disease: Current status and the future ahead. Gastroenterology 146, 1489–1499 (2014). 24560869 10.1053/j.gastro.2014.02.009 PMC4034132

[51] I. Sekirov, S. L. Russell, L. C. M. Antunes, B. B. Finlay, Gut microbiota in health and disease. Physiol. Rev. 90, 859–904 (2010). 20664075 10.1152/physrev.00045.2009

[52] J. S. Bond, G. L. Matters, S. Banerjee, R. E. Dusheck, Meprin metalloprotease expression and regulation in kidney, intestine, urinary tract infections and cancer. FEBS Lett. 579, 3317–3322 (2005). 15943977 10.1016/j.febslet.2005.03.045

[53] S. Müller, T. Schaffer, B. Flogerzi, A. Fleetwood, R. Weimann, A. M. Schoepfer, F. Seibold, Galectin-3 modulates T cell activity and is reduced in the inflamed intestinal epithelium in IBD. Inflamm. Bowel Dis. 12, 588–597 (2006). 16804396 10.1097/01.MIB.0000225341.37226.7c

[54] R. B. Sartor, Microbial influences in inflammatory bowel diseases. Gastroenterology 134, 577–594 (2008). 18242222 10.1053/j.gastro.2007.11.059

[55] M. R. Kudelka, S. R. Stowell, R. D. Cummings, A. S. Neish, Intestinal epithelial glycosylation in homeostasis and gut microbiota interactions in IBD. Nat. Rev. Gastroenterol. Hepatol. 17, 597–617 (2020). 32710014 10.1038/s41575-020-0331-7 PMC8211394

[56] E. Vazeille, M.-A. Bringer, A. Gardarin, C. Chambon, C. Becker-Pauly, S. L. Pender, C. Jakob, S. Müller, D. Lottaz, A. Darfeuille-Michaud, Role of meprins to protect ileal mucosa of Crohn's disease patients from colonization by adherent-invasive *E. coli*. PLOS ONE 6, e21199 (2011). 21698174 10.1371/journal.pone.0021199 PMC3116889

[57] N. Fortelny, S. Yang, P. Pavlidis, P. F. Lange, C. M. Overall, Proteome TopFIND 3.0 with TopFINDer and PathFINDer: Database and analysis tools for the association of protein termini to pre- and post-translational events. Nucleic Acids Res. 43, D290–D297 (2015). 25332401 10.1093/nar/gku1012 PMC4383881

[58] G. R. Vasta, Galectins as pattern recognition receptors: Structure, function, and evolution. Adv. Exp. Med. Biol. 2012, 21–36 (2012). 10.1007/978-1-4614-0106-3_2 PMC3429938 21948360

[59] S. Sciacchitano, L. Lavra, A. Morgante, A. Ulivieri, F. Magi, G. P. De Francesco, C. Bellotti, L. B. Salehi, A. Ricci, Galectin-3: One molecule for an alphabet of diseases, from a to Z. Int. J. Mol. Sci. 19, 379 (2018). 29373564 10.3390/ijms19020379 PMC5855601

[60] M. L. Fermino, C. D. Polli, K. A. Toledo, F.-T. Liu, D. K. Hsu, M. C. Roque-Barreira, G. Pereira-da-Silva, E. S. Bernardes, L. Halbwachs-Mecarelli, LPS-induced galectin-3 oligomerization results in enhancement of neutrophil activation. PLOS ONE 6, e26004 (2011). 22031821 10.1371/journal.pone.0026004 PMC3198732

[61] C. M. John, G. A. Jarvis, K. V. Swanson, H. Leffler, M. D. Cooper, M. E. Huflejt, J. M. Griffiss, Galectin-3 binds lactosaminylated lipooligosaccharides from Neisseria gonorrhoeae and is selectively expressed by mucosal epithelial cells that are infected. Cell. Microbiol. 4, 649–662 (2002). 12366402 10.1046/j.1462-5822.2002.00219.x

[62] A. Lepur, E. Salomonsson, U. J. Nilsson, H. Leffler, Ligand induced galectin-3 protein self-association. J. Biol. Chem. 287, 21751–21756 (2012). 22549776 10.1074/jbc.C112.358002 PMC3381137

[63] N. Suthahar, W. C. Meijers, H. H. W. Sillje, J. E. Ho, F. T. Liu, R. A. de Boer, Galectin-3 activation and inhibition in heart failure and cardiovascular disease: An update. Theranostics 8, 593–609 (2018). 29344292 10.7150/thno.22196 PMC5771079

[64] B. Birdsall, J. Feeney, I. D. Burdett, S. Bawumia, E. A. Barboni, R. C. Hughes, NMR solution studies of hamster galectin-3 and electron microscopic visualization of surface-adsorbed complexes: Evidence for interactions between the N- and C-terminal domains. Biochemistry 40, 4859–4866 (2001). 11294654 10.1021/bi002907f

[65] L. P. Norman, W. Jiang, X. Han, T. L. Saunders, J. S. Bond, Targeted disruption of the meprin β gene in mice leads to underrepresentation of knockout mice and changes in renal gene expression profiles. Mol. Cell. Biol. 23, 1221–1230 (2003). 12556482 10.1128/MCB.23.4.1221-1230.2003 PMC141138

[66] M. Karimova, O. Baker, A. Camgoz, R. Naumann, F. Buchholz, K. Anastassiadis, A single reporter mouse line for Vika, Flp, dre, and Cre-recombination. Sci. Rep. 8, 14453 (2018). 30262904 10.1038/s41598-018-32802-7 PMC6160450

[67] F. el Marjou, K.-P. Janssen, B. Hung-Junn Chang, M. Li, V. Hindie, L. Chan, D. Louvard, P. Chambon, D. Metzger, S. Robine, Tissue-specific and inducible Cre-mediated recombination in the gut epithelium. Genesis 39, 186–193 (2004). 15282745 10.1002/gene.20042

[68] L. Chen, Y. Shan, Y. Weng, Z. Sui, X. Zhang, Z. Liang, L. Zhang, Y. Zhang, Hydrophobic tagging-assisted N-termini enrichment for in-depth N-terminome analysis. Anal. Chem. 88, 8390–8395 (2016). 27532682 10.1021/acs.analchem.6b02453

[69] A. Takemori, P. T. Kaulich, L. Cassidy, N. Takemori, A. Tholey, Size-based proteome fractionation through polyacrylamide gel electrophoresis combined with LC–FAIMS–MS for in-depth top-down proteomics. Anal. Chem. 94, 12815–12821 (2022). 36069571 10.1021/acs.analchem.2c02777

[70] C. Broder, C. Becker-Pauly, The metalloproteases meprin α and meprin β: Unique enzymes in inflammation, neurodegeneration, cancer and fibrosis. Biochem. J. 450, 253–264 (2013). 23410038 10.1042/BJ20121751 PMC3573791

[71] C. Becker-Pauly, O. Barré, O. Schilling, U. auf dem Keller, A. Ohler, C. Broder, A. Schütte, R. Kappelhoff, W. Stöcker, C. M. Overall, Proteomic analyses reveal an acidic prime side specificity for the astacin metalloprotease family reflected by physiological substrates. Mol. Cell. Proteomics 10, M111.009233 (2011). 10.1074/mcp.M111.009233 PMC3186203 21693781

[72] A. Trad, N. Hedemann, M. Shomali, V. Pawlak, J. Grötzinger, I. Lorenzen, Development of sandwich ELISA for detection and quantification of human and murine a disintegrin and metalloproteinase17. J. Immunol. Methods 371, 91–96 (2011). 21726562 10.1016/j.jim.2011.06.015

[73] E. Gasteiger, C. Hoogland, A. Gattiker, M. R. Wilkins, R. D. Appel, A. Bairoch, in *Protein Identification and Analysis Tools on the Expasy Server*, in *The Proteomics Protocols Handbook*, J. M. Walker, Ed. (Humana Press, 2005), pp. 571–607.

[74] E. E. L. Nyström, B. Martinez-Abad, L. Arike, G. M. H. Birchenough, E. B. Nonnecke, P. A. Castillo, F. Svensson, C. L. Bevins, G. C. Hansson, M. E. Johansson, An intercrypt subpopulation of goblet cells is essential for colonic mucus barrier function. Science 372, eabb1590 (2021). 33859001 10.1126/science.abb1590 PMC8542866

[75] J. K. Volk, E. E. Nyström, S. van der Post, B. M. Abad, B. O. Schroeder, Å. Johansson, F. Svensson, S. Jäverfelt, M. E. Johansson, G. C. Hansson, G. M. H. Birchenough, The Nlrp6 inflammasome is not required for baseline colonic inner mucus layer formation or function. J. Exp. Med. 216, 2602–2618 (2019). 31420376 10.1084/jem.20190679 PMC6829596

[76] J. Aguilera-Lizarraga, M. V. Florens, M. F. Viola, P. Jain, L. Decraecker, I. Appeltans, M. Cuende-Estevez, N. Fabre, K. Van Beek, E. Perna, D. Balemans, N. Stakenborg, S. Theofanous, G. Bosmans, S. U. Mondelaers, G. Matteoli, S. I. Martínez, C. Lopez-Lopez, J. Jaramillo-Polanco, K. Talavera, Y. A. Alpizar, T. B. Feyerabend, H.-R. Rodewald, R. Farre, F. A. Redegeld, J. Si, J. Raes, C. Breynaert, R. Schrijvers, C. Bosteels, B. N. Lambrecht, S. D. Boyd, R. A. Hoh, D. Cabooter, M. Nelis, P. Augustijns, S. Hendrix, J. Strid, R. Bisschops, D. E. Reed, S. J. Vanner, A. Denadai-Souza, M. M. Wouters, G. E. Boeckxstaens, Local immune response to food antigens drives meal-induced abdominal pain. Nature 590, 151–156 (2021). 33442055 10.1038/s41586-020-03118-2 PMC7610810

[77] Y. Perez-Riverol, J. Bai, C. Bandla, D. García-Seisdedos, S. Hewapathirana, S. Kamatchinathan, D. J. Kundu, A. Prakash, A. Frericks-Zipper, M. Eisenacher, M. Walzer, S. Wang, A. Brazma, J. A. Vizcaíno, The PRIDE database resources in 2022: A hub for mass spectrometry-based proteomics evidences. Nucleic Acids Res. 50, D543–D552 (2022). 34723319 10.1093/nar/gkab1038 PMC8728295

