# Supplementary material for: Proteolytic processing of galectin-3 by meprin metalloproteases is crucial for host-microbiome homeostasis
Source: Sci Adv. 2023 Mar 31;9(13):eadf4055. doi: 10.1126/sciadv.adf4055 (PMC10065446; doi:10.1126/sciadv.adf4055)
Supplement: Supplementary file 1 — Figs. S1 to S10 [file sciadv.adf4055_sm.pdf]

Science Advances
AAAS

Supplementary Materials for

Proteolytic processing of galectin-3 by meprin metalloproteases is crucial for host-microbiome homeostasis

Cynthia Bülck *et al.*

Corresponding author: Christoph Becker-Pauly, cbeckerpauly@biochem.uni-kiel.de

Sci. Adv. **9**, eadf4055 (2023)
DOI: 10.1126/sciadv.adf4055

This PDF file includes:

Figs. S1 to S10

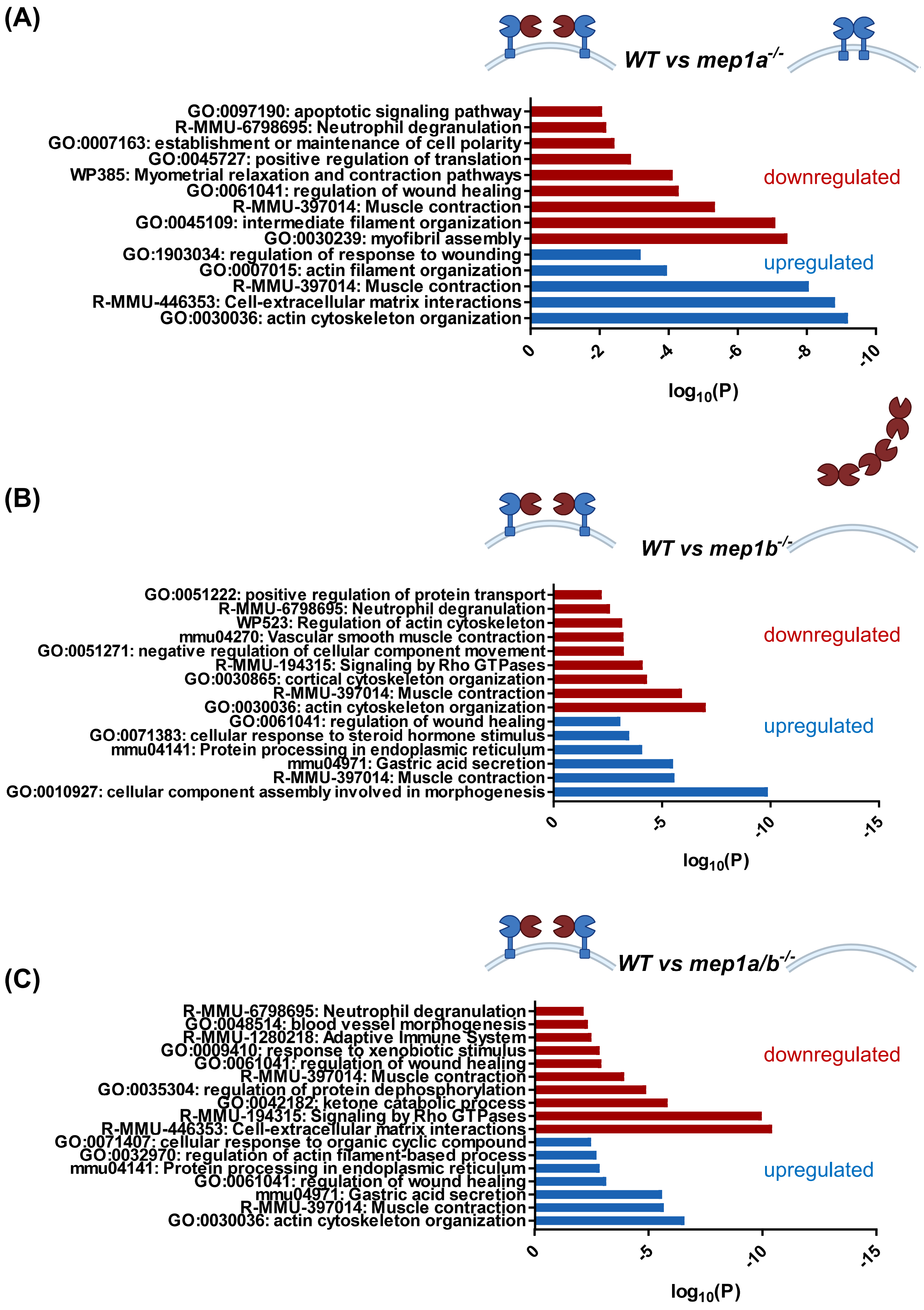
(A)
WT vs mep1a-/-
GO:0097190: apoptotic signaling pathway
R-MMU-6798695: Neutrophil degranulation
GO:0007163: establishment or maintenance of cell polarity
GO:0045727: positive regulation of translation
WP385: Myometrial relaxation and contraction pathways
GO:0061041: regulation of wound healing
R-MMU-397014: Muscle contraction
GO:0045109: intermediate filament organization
GO:0030239: myofibril assembly
GO:1903034: regulation of response to wounding
GO:0007015: actin filament organization
R-MMU-397014: Muscle contraction
R-MMU-446353: Cell-extracellular matrix interactions
GO:0030036: actin cytoskeleton organization
downregulated
upregulated
0
-2
-4
-6
-8
-10
log10(P)
(B)
WT vs mep1b-/-
GO:0051222: positive regulation of protein transport
R-MMU-6798695: Neutrophil degranulation
WP523: Regulation of actin cytoskeleton
mmu04270: Vascular smooth muscle contraction
GO:0051271: negative regulation of cellular component movement
R-MMU-194315: Signaling by Rho GTPases
GO:0030865: cortical cytoskeleton organization
R-MMU-397014: Muscle contraction
GO:0030036: actin cytoskeleton organization
GO:0061041: regulation of wound healing
GO:0071383: cellular response to steroid hormone stimulus
mmu04141: Protein processing in endoplasmic reticulum
mmu04971: Gastric acid secretion
R-MMU-397014: Muscle contraction
GO:0010927: cellular component assembly involved in morphogenesis
downregulated
upregulated
0
-5
-10
-15
log10(P)
(C)
WT vs mep1a/b-/-
R-MMU-6798695: Neutrophil degranulation
GO:0048514: blood vessel morphogenesis
R-MMU-1280218: Adaptive Immune System
GO:0009410: response to xenobiotic stimulus
GO:0061041: regulation of wound healing
R-MMU-397014: Muscle contraction
GO:0035304: regulation of protein dephosphorylation
GO:0042182: ketone catabolic process
R-MMU-194315: Signaling by Rho GTPases
R-MMU-446353: Cell-extracellular matrix interactions
GO:0071407: cellular response to organic cyclic compound
GO:0032970: regulation of actin filament-based process
mmu04141: Protein processing in endoplasmic reticulum
GO:0061041: regulation of wound healing
mmu04971: Gastric acid secretion
R-MMU-397014: Muscle contraction
GO:0030036: actin cytoskeleton organization
downregulated
upregulated
0
-5
-10
-15
log10(P)

Figure S1. Metascape analysis of proteolytic cleavage events identified by mass spectrometry-based HYTANE analysis from mouse colon

(A)-(C) Metascape was used for pathway enrichment analysis and protein complexes of the top 25 up- and downregulated proteolytic peptides identified via HYTANE analysis in wildtype mice in comparison to meprin α knockout mice (*mep1a*$^{-/-}$) (**A)**, meprin β knockout (*mep1b*$^{-/-}$) (**B)** or meprin α and meprin β double knockout (*mep1a/b*$^{-/-}$) mice **(C)**.

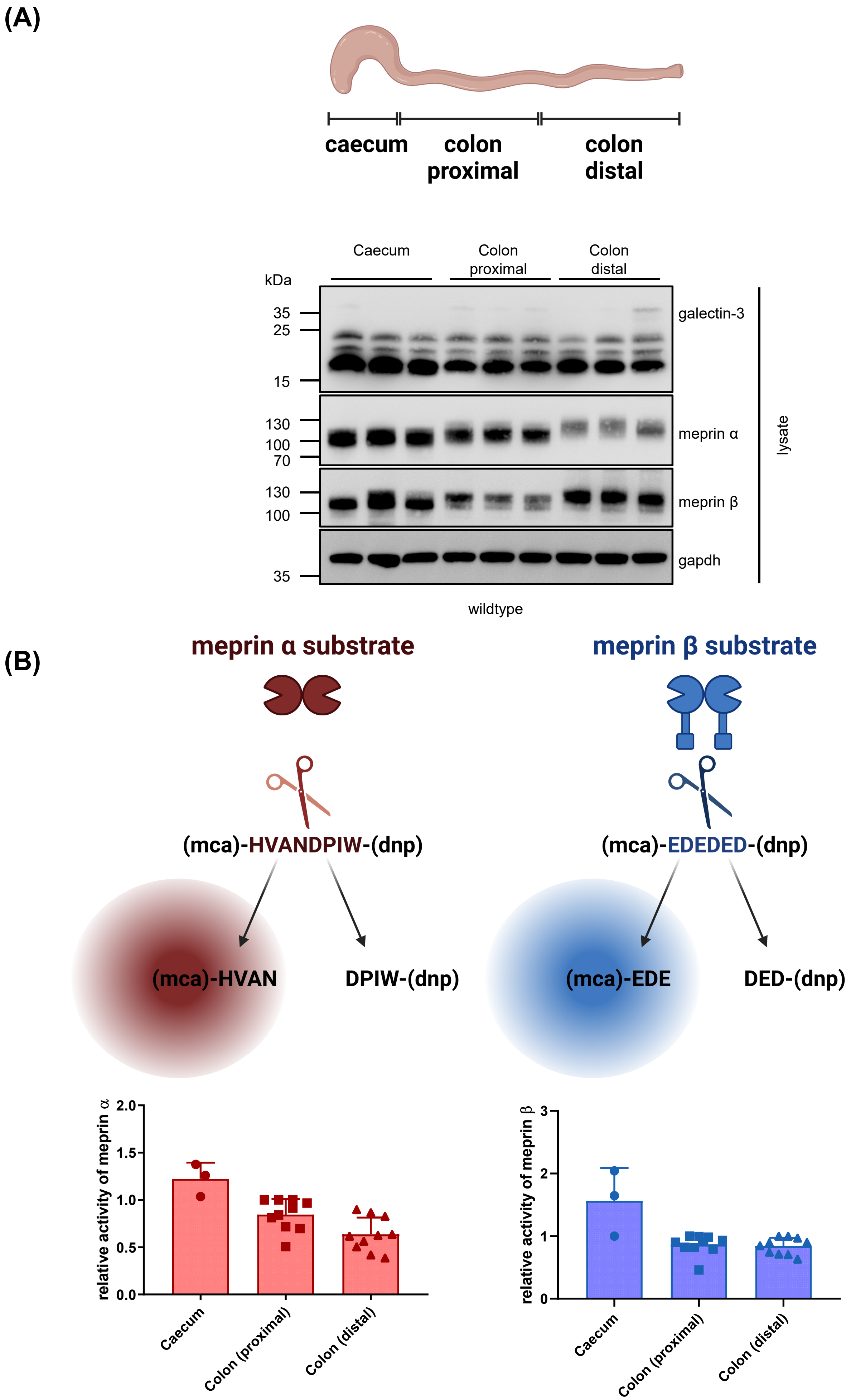
(A)
caecum
colon proximal
colon distal
Caecum
Colon proximal
Colon distal
kDa
35
25
15
130
100
70
130
100
35
galectin-3
meprin α
meprin β
gapdh
lysate
wildtype
(B)
meprin α substrate
meprin β substrate
(mca)-HVANDPIW-(dnp)
(mca)-EDEDED-(dnp)
(mca)-HVAN
DPIW-(dnp)
(mca)-EDE
DED-(dnp)
relative activity of meprin α
2.0
1.5
1.0
0.5
0.0
Caecum
Colon (proximal)
Colon (distal)
relative activity of meprin β
3
2
1
0
Caecum
Colon (proximal)
Colon (distal)

Figure S2. Proteolytic activity and cleavage of galectin-3 in caecum, proximal and distal colon.

(A) Galectin-3 cleavage in tissue lysates from caecum, proximal and distal colon from three biological replicates of wildtype mice analyzed by Western-Blot analysis using specific galectin-3, meprin α and meprin β antibodies. Gapdh served as loading control. **(B)** Proteolytic activity of meprin α (left) and meprin β (right) in tissue lysates from the caecum (n=3), proximal (n=10) and distal colon (n=10). Data are represented as mean ± SD.

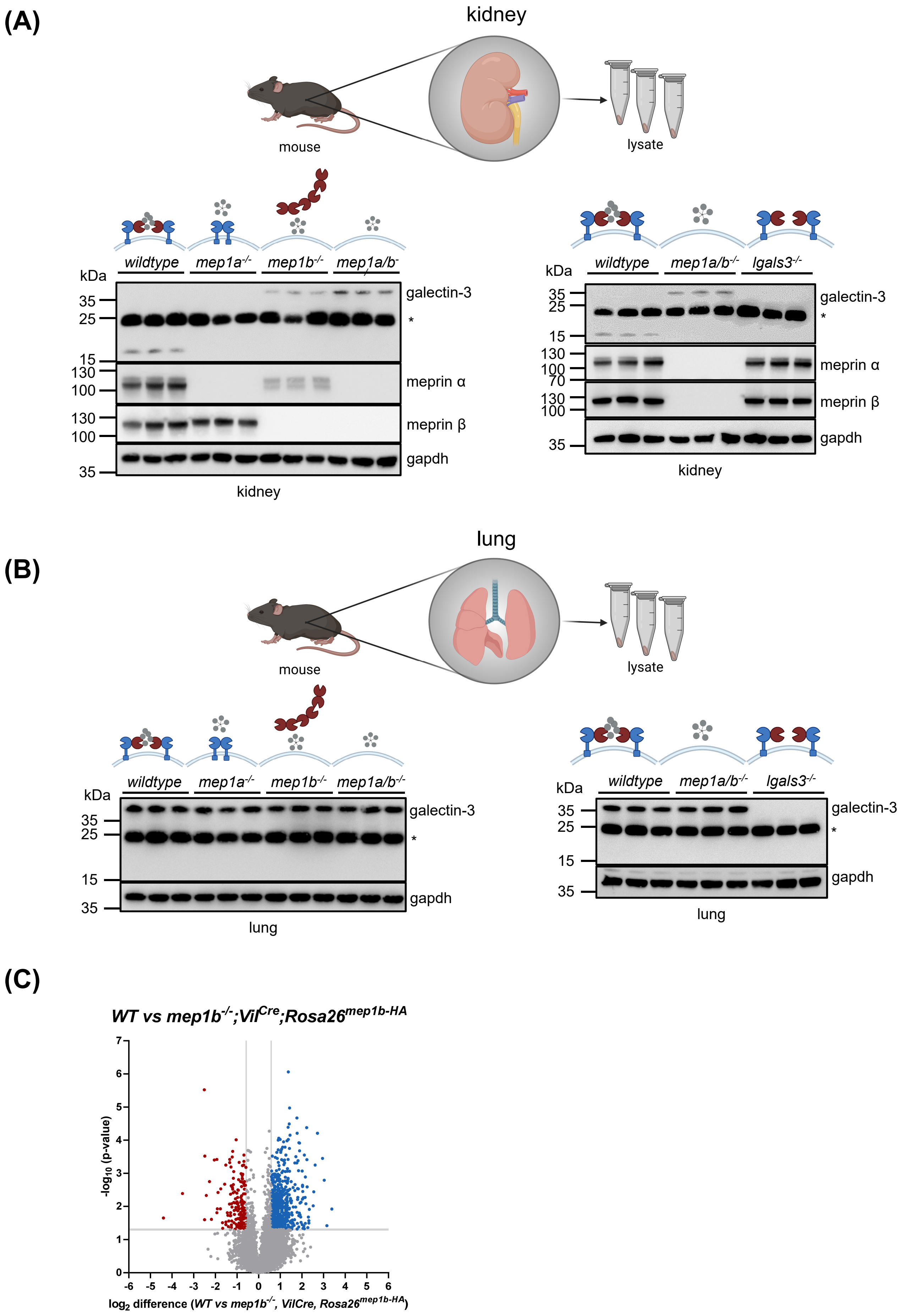
(A)
kidney
mouse
lysate
wildtype
mep1a-/-
mep1b-/-
mep1a/b-/-
kDa
35
25
15
130
100
galectin-3
*
meprin α
meprin β
gapdh
kidney
wildtype
mep1a/b-/-
lgals3-/-
kDa
35
25
15
130
100
70
130
100
35
galectin-3
*
meprin α
meprin β
gapdh
kidney
(B)
lung
mouse
lysate
wildtype
mep1a-/-
mep1b-/-
mep1a/b-/-
kDa
35
25
15
35
galectin-3
*
gapdh
lung
wildtype
mep1a/b-/-
lgals3-/-
kDa
35
25
15
35
galectin-3
*
gapdh
lung
(C)
WT vs mep1b-/-;VilCre;Rosa26mep1b-HA
-log10 (p-value)
0
1
2
3
4
5
6
7
-6
-5
-4
-3
-2
-1
0
1
2
3
4
5
6
log2 difference (WT vs mep1b-/-, VilCre, Rosa26mep1b-HA)

Figure S3. *In vivo* proteolytic processing of galectin-3 in different organs is dependent on meprin expression

(A)-(B) Analysing galectin-3 cleavage and meprin expression in kidney (**A)** or lung (**B)** tissue from three biological replicates of wildtype, *mep1a*$^{-/-}$, *mep1b*$^{-/-}$ or *mep1a/b*$^{-/-}$ mice (left) and *lgals3*$^{-/-}$ mice (right) via Western-Blot analysis. * marks an unspecific band. **(C)** Volcano plots showing all identified proteolytic events detected following HYTANE analysis of wildtype mice in comparison to *mep1b*$^{-/-}$*;Vil*^{Cre};*Rosa26*$^{mep1b\text{-}HA}$ mice. Grey lines represent threshold values (±0.58 for $\log_2$ difference and p=0.05). No alterations in abundance of the proteolytic fragments of galectin-3 could be observed.

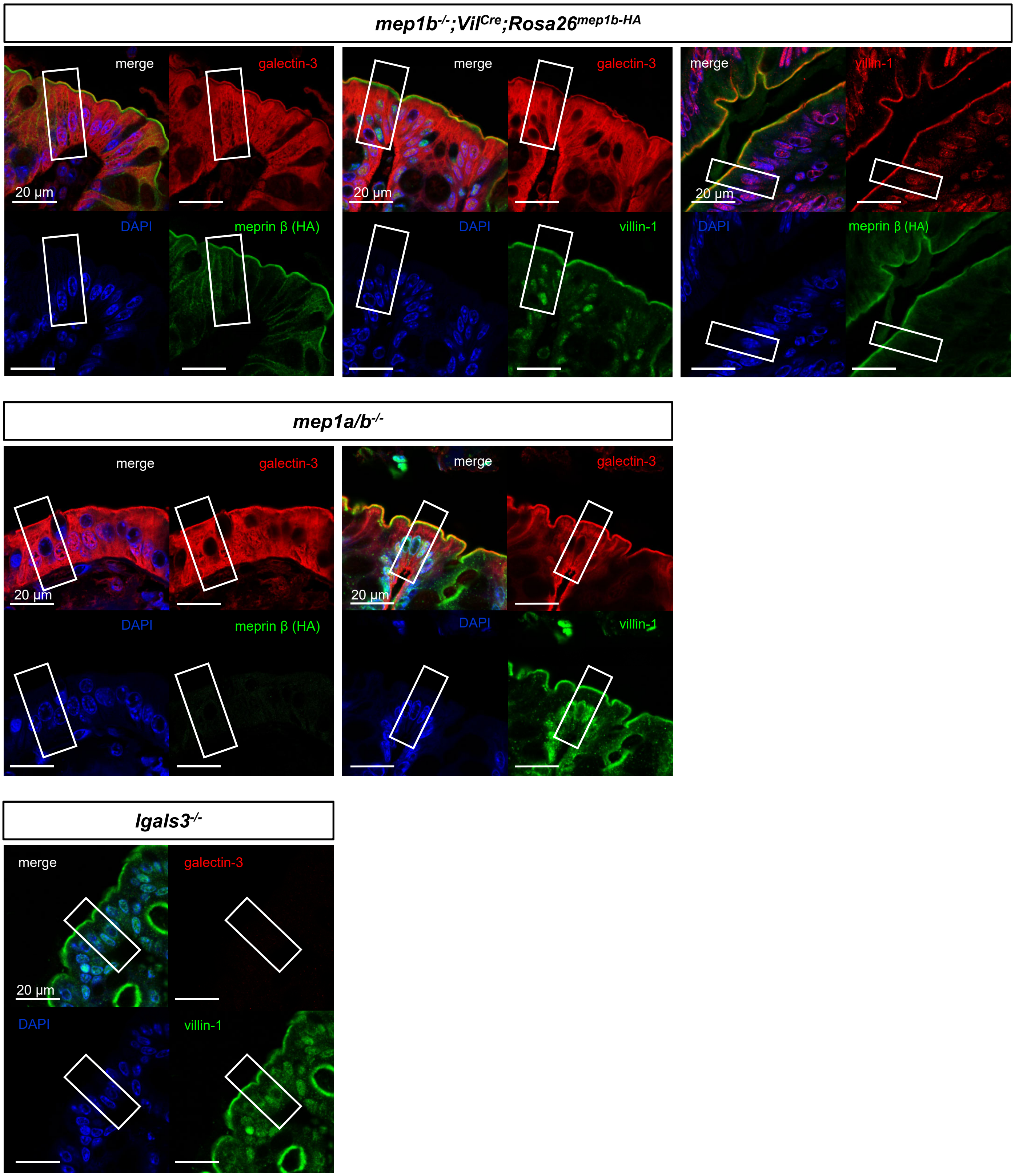

mep1b-/-;VilCre;Rosa26mep1b-HA
merge
galectin-3
20 µm
DAPI
meprin β (HA)
villin-1
mep1a/b-/-
lgals3-/-

Figure S4. Tissue distribution of galectin-3 in mouse colon

Confocal microscopy of intestinal Swiss roles from the whole colon of either *mep1b*$^{-/-}$;*Vil*^{Cre};*Rosa26*$^{mep1b\text{-}HA}$ mice, *mep1a/b*$^{-/-}$ mice or *lgals3*$^{-/-}$ mice. Tissues were stained against galectin-3 (red), meprin β (green) or villin-1 (red or green). For re-expressed HA-tagged meprin β the HA-antibody was used. Nuclear staining was visualized using 4′,6-diamidino-2-phenylindole (DAPI) (blue). Boxes indicate areas of higher magnification pictures shown in Figure 2C. Scale bars: 20 µm.

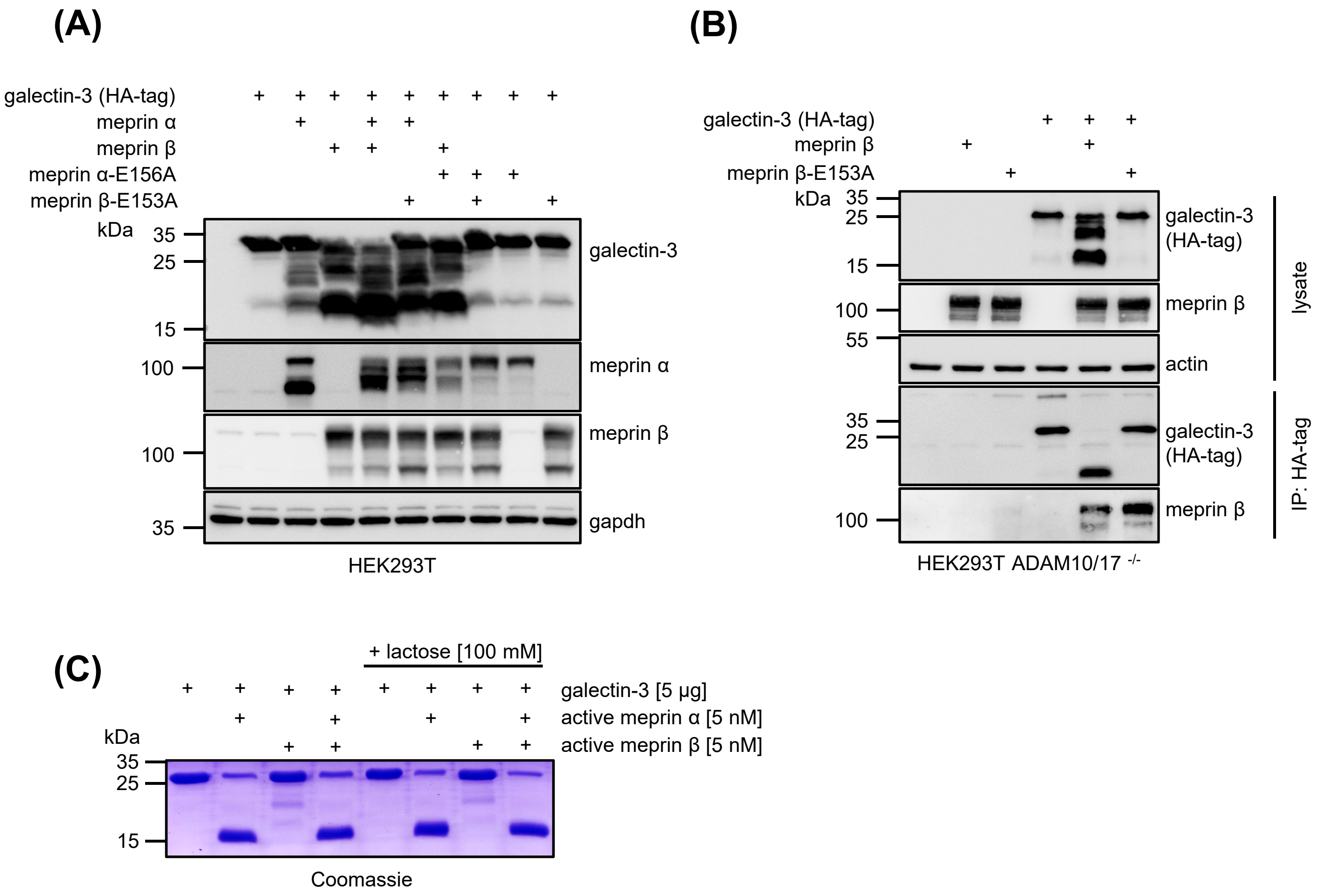

Figure S5. Cleavage and interaction of galectin-3 with inactive variants of meprin metalloproteases in human HEK293T cells and analysis of recombinant galectin-3 cleavage in the presence of lactose

(A) HEK ADAM10/17$^{-/-}$ cells were transfected with galectin-3 and either wildtype meprin β or a catalytically inactive variant (meprin β-E153A). After cell lysis, co-immunoprecipitation was performed using a HA-tag antibody against galectin-3 C-terminus. Lysate controls and immunoprecipitates were analyzed by Western-Blot. (**B)** Analysing the alterations in galectin-3 cleavage by co-expression of galectin-3 with meprin α and/or meprin β as well as catalytically inactive variants of meprin α (meprin α-E165A) and meprin β (meprin β-E153A) in HEK293T cells via immunoblotting. **(C)** Recombinant human (20 µg) galectin-3 was incubated with recombinant human active meprin α (5 nM) and/or meprin β (5 nM) as well as treated with 100 mM lactose for 2 h at 37°C. The proteolytic processing of galectin-3 was analyzed by SDS-PAGE and subsequent Coomassie brilliant blue staining.

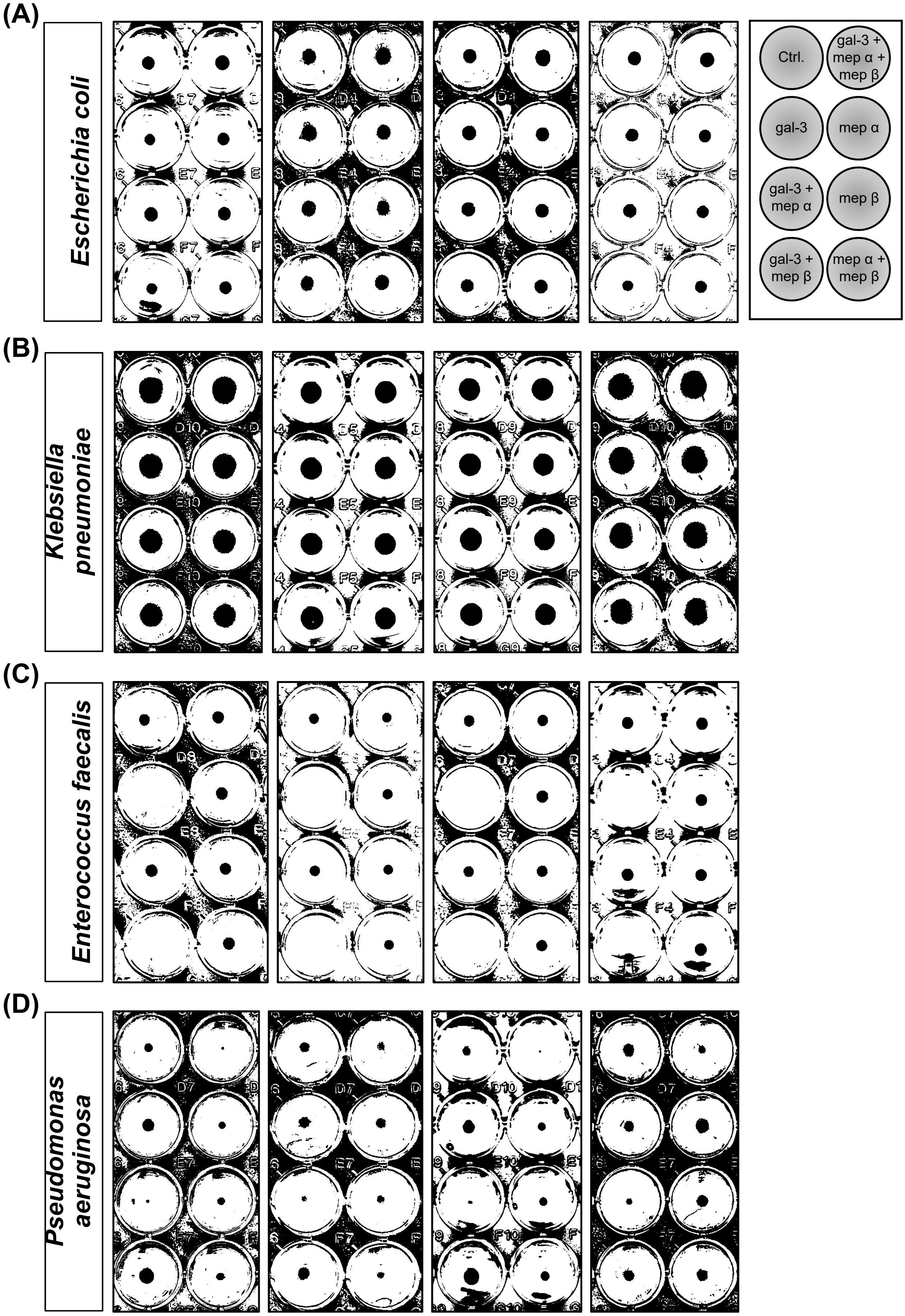
(A)
Escherichia coli
Ctrl.
gal-3 + mep α + mep β
gal-3
mep α
gal-3 + mep α
mep β
gal-3 + mep β
mep α + mep β
(B)
Klebsiella pneumoniae
(C)
Enterococcus faecalis
(D)
Pseudomonas aeruginosa

Figure S6. Pictures of bacterial agglutination assays in the presence or absence of galectin-3 and meprin proteases.

(A)-(D) *Escherichia coli* (**A**), *Klebsiella pneumoniae* (**B**), *Enterococcus faecalis* **(C)** and *Pseudomonas aeruginosa* **(D)** were incubated with recombinant galectin-3 (gal-3) or galectin-3 pre-incubated with 5 nM active recombinant meprin α (mep α) and/or meprin β (mep β) for 24 hours and the presence or absence of bacterial agglutination was examined. As a negative control (Ctrl.) buffer (20 mM HEPES, pH 7.2) or active recombinant meprins were used. Area of non-agglutinated bacteria was calculated using ImageJ Fiji. All values were normalized to the control (HEPES buffer), while the control corresponds to 0 and maximum agglutination to 1.

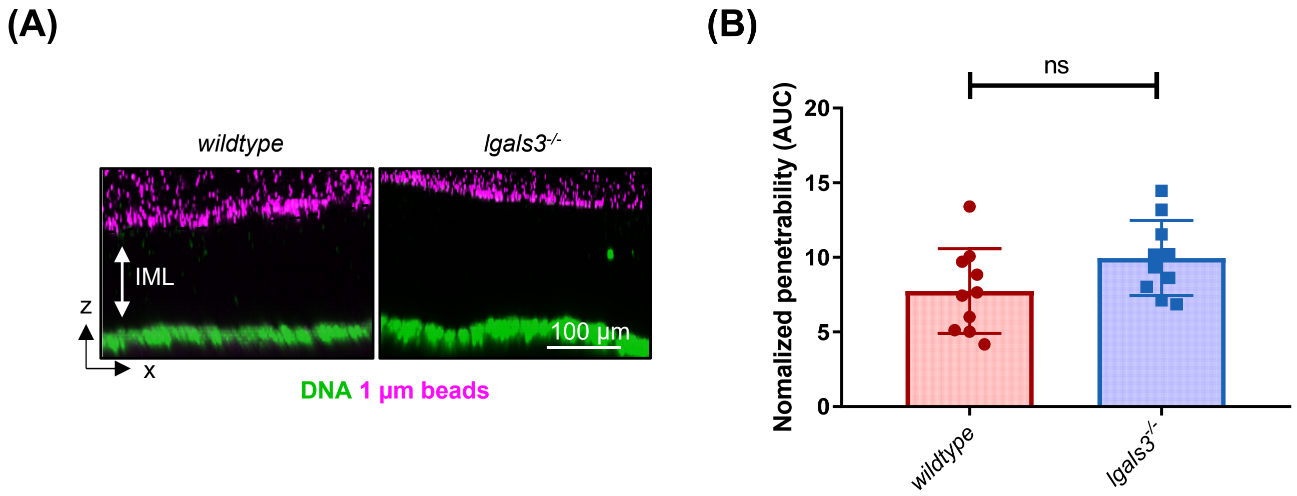

Figure S7. *Ex vivo* mucus measurements revealed no alterations in colonic mucus penetrability in colon of *lgals3$^{-/-}$* versus wildtype mice

(A) For mucus penetrability measurements distal colon tissue of wildtype and *lgals3$^{-/-}$* (n=10) mice was mounted in a horizontal chamber and fluorescent bacterial-size beads were added on the explant. Beads were allowed to sediment for 5 min before the distribution was visualized using confocal microscopy. Images are representative confocal z-stack projections obtained from the experiments (Scale bars, 100 µm). Green: DNA (stained with Syto9); pink: beads; IML: inner mucus layer. (**B**) Quantification of inner mucus layer (IML) penetrability of wildtype and *lgals3$^{-/-}$* mice based on data shown in (A). AUC; area under curve.

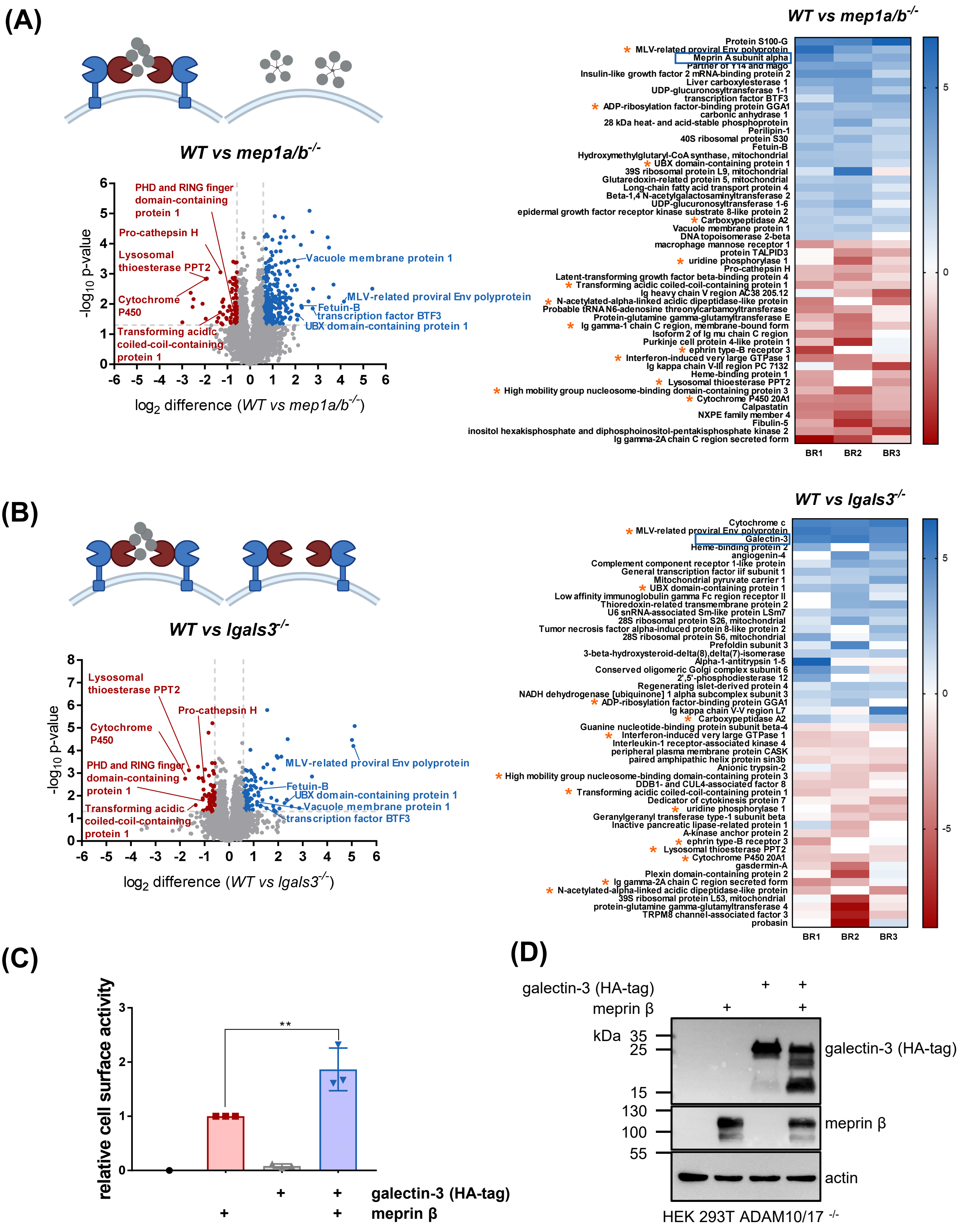
(A)
WT vs mep1a/b-/-
6
5
4
3
2
1
0
-log10 p-value
-6 -5 -4 -3 -2 -1 0 1 2 3 4 5 6
log2 difference (WT vs mep1a/b-/-)
PHD and RING finger domain-containing protein 1
Pro-cathepsin H
Lysosomal thioesterase PPT2
Cytochrome P450
Transforming acidic coiled-coil-containing protein 1
Vacuole membrane protein 1
MLV-related proviral Env polyprotein
Fetuin-B
transcription factor BTF3
UBX domain-containing protein 1
WT vs mep1a/b-/-
Protein S100-G
MLV-related proviral Env polyprotein
Meprin A subunit alpha
Partner of Y14 and mago
Insulin-like growth factor 2 mRNA-binding protein 2
Liver carboxylesterase 1
UDP-glucuronosyltransferase 1-1
transcription factor BTF3
ADP-ribosylation factor-binding protein GGA1
carbonic anhydrase 1
28 kDa heat- and acid-stable phosphoprotein
Perilipin-1
40S ribosomal protein S30
Fetuin-B
Hydroxymethylglutaryl-CoA synthase, mitochondrial
UBX domain-containing protein 1
39S ribosomal protein L9, mitochondrial
Glutaredoxin-related protein 5, mitochondrial
Long-chain fatty acid transport protein 4
Beta-1,4 N-acetylgalactosaminyltransferase 2
UDP-glucuronosyltransferase 1-6
epidermal growth factor receptor kinase substrate 8-like protein 2
Carboxypeptidase A2
Vacuole membrane protein 1
DNA topoisomerase 2-beta
macrophage mannose receptor 1
protein TALPID3
uridine phosphorylase 1
Pro-cathepsin H
Latent-transforming growth factor beta-binding protein 4
Transforming acidic coiled-coil-containing protein 1
Ig heavy chain V region AC38 205.12
N-acetylated-alpha-linked acidic dipeptidase-like protein
Probable tRNA N6-adenosine threonylcarbamoyltransferase
Protein-glutamine gamma-glutamyltransferase E
Ig gamma-1 chain C region, membrane-bound form
Isoform 2 of Ig mu chain C region
Purkinje cell protein 4-like protein 1
ephrin type-B receptor 3
Interferon-induced very large GTPase 1
Ig kappa chain V-III region PC 7132
Heme-binding protein 1
Lysosomal thioesterase PPT2
High mobility group nucleosome-binding domain-containing protein 3
Cytochrome P450 20A1
Calpastatin
NXPE family member 4
Fibulin-5
inositol hexakisphosphate and diphosphoinositol-pentakisphosphate kinase 2
Ig gamma-2A chain C region secreted form
BR1 BR2 BR3
5
0
(B)
WT vs lgals3-/-
8
7
6
5
4
3
2
1
0
-log10 p-value
-6 -5 -4 -3 -2 -1 0 1 2 3 4 5 6
log2 difference (WT vs lgals3-/-)
Lysosomal thioesterase PPT2
Pro-cathepsin H
Cytochrome P450
PHD and RING finger domain-containing protein 1
Transforming acidic coiled-coil-containing protein 1
MLV-related proviral Env polyprotein
Fetuin-B
UBX domain-containing protein 1
Vacuole membrane protein 1
transcription factor BTF3
WT vs lgals3-/-
Cytochrome c
MLV-related proviral Env polyprotein
Galectin-3
Heme-binding protein 2
angiogenin-4
Complement component receptor 1-like protein
General transcription factor IIF subunit 1
Mitochondrial pyruvate carrier 1
UBX domain-containing protein 1
Low affinity immunoglobulin gamma Fc region receptor II
Thioredoxin-related transmembrane protein 2
U6 snRNA-associated Sm-like protein LSm7
28S ribosomal protein S26, mitochondrial
Tumor necrosis factor alpha-induced protein 8-like protein 2
28S ribosomal protein S6, mitochondrial
Prefoldin subunit 3
3-beta-hydroxysteroid-delta(8),delta(7)-isomerase
Alpha-1-antitrypsin 1-5
Conserved oligomeric Golgi complex subunit 6
2',5'-phosphodiesterase 12
Regenerating islet-derived protein 4
NADH dehydrogenase [ubiquinone] 1 alpha subcomplex subunit 3
ADP-ribosylation factor-binding protein GGA1
Ig kappa chain V-V region L7
Carboxypeptidase A2
Guanine nucleotide-binding protein subunit beta-4
Interferon-induced very large GTPase 1
Interleukin-1 receptor-associated kinase 4
peripheral plasma membrane protein CASK
paired amphipathic helix protein sin3b
Anionic trypsin-2
High mobility group nucleosome-binding domain-containing protein 3
DDB1- and CUL4-associated factor 8
Transforming acidic coiled-coil-containing protein 1
Dedicator of cytokinesis protein 7
uridine phosphorylase 1
Geranylgeranyl transferase type-1 subunit beta
Inactive pancreatic lipase-related protein 1
A-kinase anchor protein 2
ephrin type-B receptor 3
Lysosomal thioesterase PPT2
Cytochrome P450 20A1
gasdermin-A
Plexin domain-containing protein 2
Ig gamma-2A chain C region secreted form
N-acetylated-alpha-linked acidic dipeptidase-like protein
39S ribosomal protein L53, mitochondrial
protein-glutamine gamma-glutamyltransferase 4
TRPM8 channel-associated factor 3
probasin
BR1 BR2 BR3
5
0
-5
(C)
relative cell surface activity
3
2
1
0
**
galectin-3 (HA-tag)
meprin β
(D)
galectin-3 (HA-tag)
meprin β
kDa 35
25
15
130
100
55
galectin-3 (HA-tag)
meprin β
actin
HEK 293T ADAM10/17 -/-

Figure S8. Pre-N-terminomics and cell surface activity assay revealed an interaction of meprins with galectin-3 on transcriptional as well as on protein level

(A)-(B) Volcano plots (left) showing all identified proteins detected following protein label free quantification of wildtype mice in comparison to *mep1a/b*$^{-/-}$ (**A**) or *lgals3*$^{-/-}$ mice (**B**). Grey lines represent threshold values (±0.58 for $\log_2$ difference and p=0.05). Proteins that were significantly abundant in both approaches were labelled in red and blue. Heatmaps (right) of the top 25 highest (blue) and less (red) abundant proteolytic peptides from three biological replicates (BR) (blue = wildtype > knockout; red = wildtype < knockout; sorted by $\log_2$ difference). All proteins, that were identified in both approaches are highlighted with an orange star. **(C)** Cell surface activity of meprin β on HEK293T ADAM10/17$^{-/-}$ cells upon co-transfection with galectin-3 (n=3). Data are represented as mean ± SD and statistical analysis was assessed by one-way ANOVA followed by a Tukey post-test (*: p<0.05; **: p<0.01; ***: p<0.001). (**D**) Representative Western blot of a successful transfection in **(C)**.

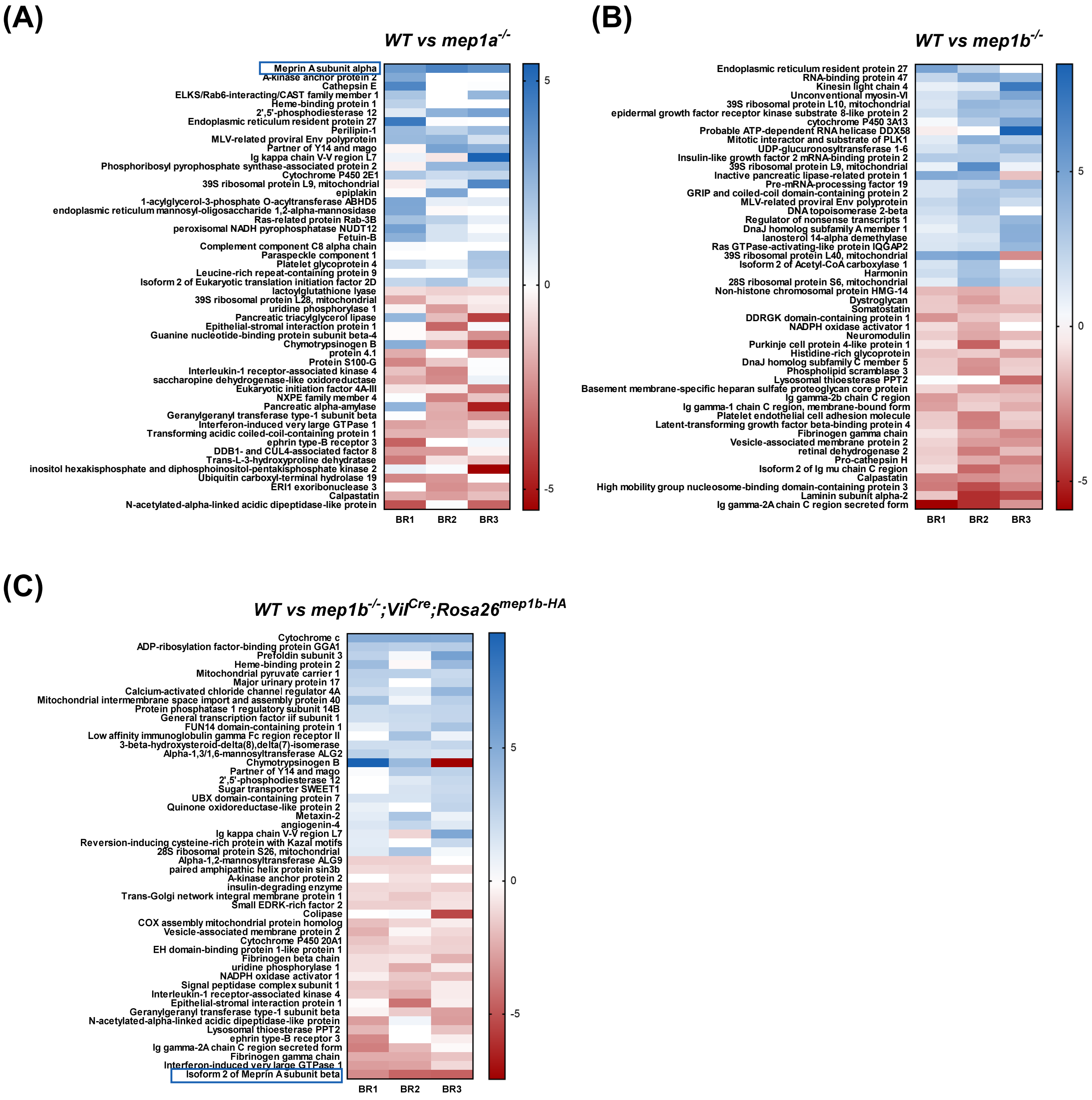

Figure S9. Heatmaps of the protein label free quantification data between wildtype and *mep1a*$^{-/-}$, *mep1b*$^{-/-}$ or *mep1b*$^{-/-}$;*Vil*^{Cre};Rosa26$^{mep1b\text{-}HA}$ mice.

(A)-(C) Heatmaps of the top 25 highest (blue) and less (red) abundant proteins from three biological replicates (BR) (blue = wildtype > knockout; red = wildtype < knockout; sorted by $\log_2$ difference). Comparison of wildtype versus *mep1a*$^{-/-}$ mice (**A**), *mep1b*$^{-/-}$ mice (**B**) or *mep1b*$^{-/-}$;*Vil*^{Cre};*Rosa26*$^{mep1b\text{-}HA}$ mice **(C)**.

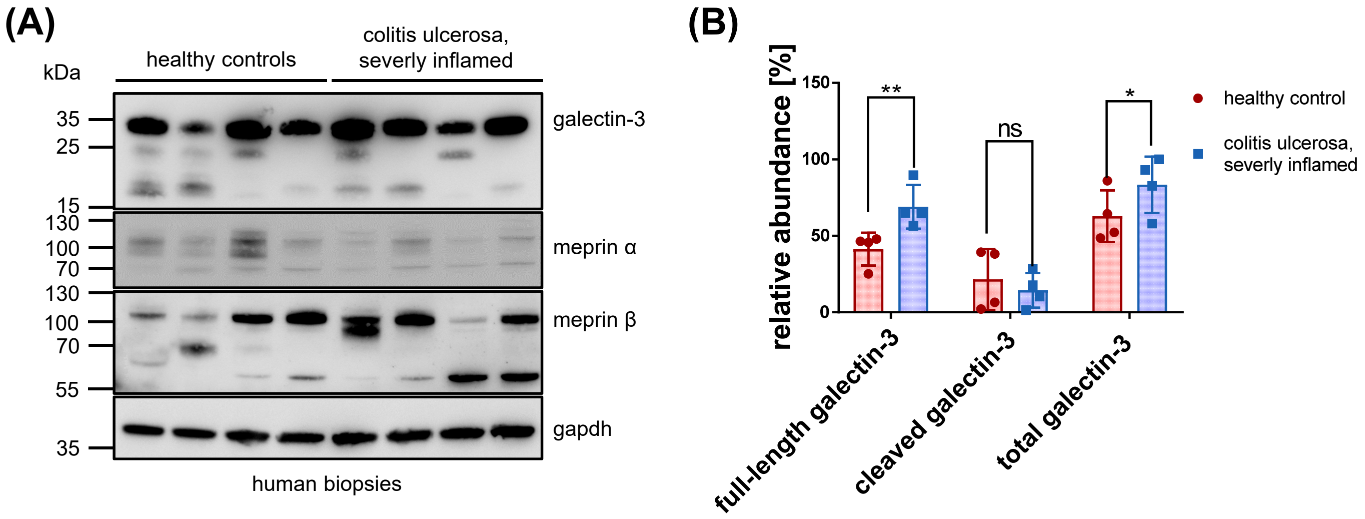

Figure S10. Full-length galectin-3 is increased in human biopsies of colonic tissue from patients with ulcerative colitis.

(A) Western-Blot analysis of heathy controls and severely inflamed human colonic biopsies derived from ulcerative colitis patient's using an antibody specific for galectin-3, human meprin α and human meprin β (n=4). (**B)** Densitometric analysis of full-length and cleaved galectin-3 based on the total amount of galectin-3 calculated by ImageJ from four biological replicates as shown in (A). Data are represented as mean ± SD and statistical analysis was assessed by two-way ANOVA followed by a Tukey post-test (*: $p<0.05$; **: $p<0.01$; ***: $p<0.001$).
